# Comprehensive discovery of novel structured noncoding RNAs in 26 bacterial genomes

**DOI:** 10.1080/15476286.2021.1917891

**Published:** 2021-05-10

**Authors:** Kenneth I. Brewer, Etienne B. Greenlee, Gadareth Higgs, Diane Yu, Gayan Mirihana Arachchilage, Xi Chen, Nicholas King, Neil White, Ronald R. Breaker

**Affiliations:** a Department of Molecular Biophysics and Biochemistry, Yale University, New Haven, CT, USA; b Department of Molecular, Cellular and Developmental Biology, Yale University, New Haven, CT, USA; c Howard Hughes Medical Institute, Yale University, New Haven, CT, USA

**Keywords:** Comparative sequence analysis, NAD+, regulatory RNA, riboswitch, uORF

## Abstract

Comparative sequence analysis methods are highly effective for uncovering novel classes of structured noncoding RNAs (ncRNAs) from bacterial genomic DNA sequence datasets. Previously, we developed a computational pipeline to more comprehensively identify structured ncRNA representatives from individual bacterial genomes. This search process exploits the fact that genomic regions serving as templates for the transcription of structured RNAs tend to be present in longer than average noncoding ‘intergenic regions’ (IGRs) that are enriched in G and C nucleotides compared to the remainder of the genome. In the present study, we apply this computational pipeline to identify structured ncRNA candidates from 26 diverse bacterial species. Numerous novel structured ncRNA motifs were discovered, including several riboswitch candidates, one whose ligand has been identified and others that have yet to be experimentally validated. Our findings support recent predictions that hundreds of novel ribo-switch classes and other ncRNAs remain undiscovered among the limited number of bacterial species whose genomes have been completely sequenced.

## Introduction

Each bacterial species carries genes expressing structured noncoding RNAs (ncRNAs) whose nucleotide sequences and folded shapes are critical for their biological and biochemical functions. These characteristics are most prominently manifested by molecules such as tRNAs and rRNAs that collaborate with mRNAs to catalyse the synthesis of all genetically encoded polypeptides [1]. However, other classes of structured ncRNAs are likewise essential for the survival of nearly all bacterial species. These include ncRNA classes such as (i) the RNase P ribozyme [2] whose site-specific phosphoester hydrolysis activity processes many precursor RNA transcripts, (ii) the signal recognition particle RNA [3], which is instrumental in routing certain proteins to cell membranes, and (iii) 6S RNA [4], which serves as a regulator of RNA polymerase activity. Members of another class of highly folded RNA molecules, called tmRNA [5], exploit both a complex tertiary structure and a protein-coding region to simultaneously induce the release of ribosomes from broken mRNAs while tagging truncated proteins with a polypeptide sequence designating them for disposal. This list of fundamental biochemical and biological functions of bacterial ncRNA classes is made larger when including less widespread classes that also exhibit remarkable functions, including various RNA processing (ribozymes) [6,7] and gene control (riboswitches) [8–10] activities.

A focus of our research team in recent years has been the discovery of riboswitches that sense specific metabolites or inorganic ions to regulate the expression of genes whose protein products synthesize, transport, utilize, or respond to the ligands being monitored. Over 50 distinct classes of ribo-switches have been experimentally validated to date [11–23], and many of these sense nucleotide-derived compounds proposed to have been present during an era of biology before the evolutionary emergence of proteins [24–28]. Thus, uncovering additional classes of riboswitches promises to reveal more about how modern cells regulate critical biochemical processes, and perhaps also to provide insight into the functions of ancient RNA-based regulatory systems [29,30].

It has been proposed [11,30,31] that many bacterial ribo-switch classes remain hidden in the existing bacterial genomic datasets, and that these undiscovered representatives are likely to be rare compared to those from the most abundant classes that have already been experimentally validated. If true, we are in an era wherein many hundreds of sparsely represented riboswitch classes remain undiscovered in existing genomic DNA datasets. Unfortunately, this rarity creates a considerable barrier for those who seek to uncover novel riboswitch classes. Past computational search campaigns that led to the discovery of multiple novel riboswitch classes [32–36] typically have exploited comparative sequence analysis algorithms. These algorithms seek similarity in sequence and possible secondary structure features of one intergenic region (IGR) with that of all or a subset of other IGRs in the database under examination. These approaches have uncovered ribo-switch classes that are abundant and phylogenetically diverse. However, they typically fail to identify rare riboswitch candidates, particularly when only a few highly similar representatives are present in the database.

To partly overcome this problem, we developed a computational pipeline [37,38] that substantially improves the chances that rare riboswitch candidates and other structured ncRNAs will be uncovered. This ‘GC-IGR’ pipeline takes advantage of the fact that structured ncRNAs are encoded in IGRs that have unique characteristics compared to most other IGRs in certain bacterial genomes. Specifically, organisms whose genomes are rich in A and T nucleotides usually carry templates for the transcription of structured ncRNAs in IGRs that have a higher than average GC content [39,40]. Also, most IGRs are relatively short unless they carry ncRNA templates. By sorting ‘unknown’ IGRs (those that are not known to carry a genetic element) based on both length and GC content, IGRs that carry previously undiscovered ncRNA motifs are likely to cluster with IGRs that are already known to serve as templates for structured ncRNAs. By analysing the IGRs with these unique characteristics, we have been able to discover various novel classes of ncRNAs, such as riboswitches and other structured RNA and DNA motifs [37,38]. The detailed methodology employed in the GC-IGR computational pipeline has been demonstrated previously by examining the IGRs of five bacterial genomes [38]. This same methodology has now been employed herein to search for structured ncRNA motifs from an additional 26 bacterial genomes. We report the discovery of a variety of novel ncRNA motifs, which is consistent with earlier predictions [11,30,31] that numerous functional RNAs, including distinct candidate riboswitch classes, remain to be discovered in the genomes of many bacterial species.

## Results and discussion

### Genomes and IGRs selected for comparative sequence analysis

In continuation of our campaign to discover novel ncRNA motifs by using the GC-IGR bioinformatics pipeline [37,38], we analysed an additional 26 bacterial genomes (**Table S1**) that were chosen in part to expand the number of phyla examined with this method. Although genomes were selected largely from those that have favourable %GC values, our choices where somewhat arbitrary because we intend to eventually examine all genomes with characteristics suitable for analysis by this pipeline. Chosen genomes represent the following phyla: Aquificae, Chlamydiae, Firmicutes, Fusobacteria, Proteobacteria, Spirochaetes, Tenericutes, Thermodesulfobacteria, and Thermotogae. This collection of genomes expands the analysed phyla from three in the initial work (Firmicutes, Proteobacteria, and Synergistetes) to nine. Additionally, the classes of Proteobacteria were expanded from three to five. Within this collection of 26 bacterial genomes, there is substantial variability in the total number of IGRs found within a genome, in the average IGR length, and in the average IGR %GC content (**Table S1** and **Figures S1-S26**). This constitutes a broader distribution of IGR characteristics compared to those examined in the previous studies, which provides an opportunity to further evaluate the robustness of the GC-IGR pipeline.

Certain IGRs from these 26 genomes were chosen for in-depth analysis in the same manner as previously described [38]. Briefly, the IGRs of each genome were plotted using two parameters: the length of the IGR and its GC content. Genomes that exhibit substantial clustering of IGRs carrying known structured ncRNAs were favoured for further analysis. IGRs lacking a known function, called ‘unknown IGRs’, were selected for analysis if they cluster near to IGRs carrying known structured ncRNAs. To partition unknown IGRs for additional analysis from the rest, we generally sought to define a region on each plot that yielded near equal numbers of known ncRNAs and unknown IGRs. Potential structural elements residing in unknown IGRs of interest were then sought. For example, the presence of conserved nucleotide sequences between two or more IGRs is suggestive of a conserved RNA architecture. Likewise, the presence of nucleotide covariation, such that a mutation at one position always occurs in conjunction with a mutation at a second site that retains base-pairing potential, is strongly indicative of a conserved secondary structure feature.

By identifying conserved sequences and secondary structures, we can focus additional attention on the unknown IGRs that carry novel candidate ncRNA motifs. However, some of the motifs identified in this manner represent previously discovered, but misannotated or unannotated ncRNAs, such as tRNAs, riboswitches, or other structured RNAs. Similarly, some conserved features reflect the presence of conserved open reading frames (ORFs), which are identified and classified based on bioinformatic evidence of their ability to code for protein. IGRs that carry truly distinct motifs are tentatively named based on their apparent potential to function as a noncoding RNA, including riboswitch candidates whose functions might be predicted based on their gene associations and genomic orientations. Specifically, strong riboswitch candidates typically have robust sequence and structural conservation, a genomic orientation that consistently positions the motif upstream of its associated protein-coding genes, and gene associations that are indicative of regulation by a small molecule or inorganic ion ligand [11]. Importantly, each proposed function for a novel motif should be considered highly speculative and meant only to create a preliminary organization of the findings. In each instance, biochemical or genetic validation experiments are subsequently needed to confirm the proposed functions.

### Classification of newly discovered nucleic acid motifs

In a previous study [38], we defined five general categories into which we classified all IGRs examined by implementing the GC-IGR pipeline. This exercise is not intended to serve as an error-free evaluation of each motif, but rather to provide a preliminary assessment that might help researchers make decisions regarding future experimental analyses. For the current study, we follow these same guidelines to make predictions regarding the possible functions of novel motifs, which are repeated below.

(i) Unnamed: Insufficient evidence to classify.

(ii) Low-ranking candidate (LRC): Typically fewer than 5 unique representatives and a poor consensus model.

(iii) Medium-ranking candidate (MRC): Typically fewer than 20 unique representatives and/or a poor consensus model.

(iv) High-ranking candidate (HRC): Many representatives and a good consensus model, but insufficient information regarding possible function.

(v) Named candidate: Could be rare, but usually has many representatives with a good consensus model and some evidence supporting a hypothesis for function.

Some sequences annotated as IGRs in the various DNA sequence databases actually function as ORFs for ordinary-sized proteins, or serve as templates for the translation of known ncRNAs. These misannotated IGRs are removed from our candidate list before sorting the IGRs by the criteria just described. Note that category (v) includes any motifs where we judged that sufficient information was evident to predict the motif’s function, including riboswitches.

### Strong riboswitch candidates

Riboswitches are one of many types of ncRNAs that are expected to be uncovered by the GC-IGR pipeline, and these will contribute to the ‘named candidate’ category as defined above. They are also one of the more straightforward candidate ncRNA types to generate hypotheses regarding their functions. Many of the known riboswitch classes exhibit characteristics that are conspicuous due to the necessities of forming metabolite-sensing (aptamer) and gene-control (expression platform) domains. Given that there is always some degree of uncertainty when assessing whether a novel motif functions as an RNA regulatory element, in the current study, we have arranged the riboswitch candidates into strong and weak groups to give readers a general indication of the merits of each motif. Strong riboswitches candidates typically exhibit extensive sequence and structural conservation, associate with genes for related biochemical or biological processes, and are oriented in a manner that is consistent with a gene control function, among other attributes.

However, some newly found ncRNA motifs imperfectly reflect these characteristics, and therefore we consider them weak riboswitch candidates. Regardless, we recommend that researchers who wish to pursue the experimental validation of even the strong riboswitch candidates presented herein should proceed with appropriate scepticism regarding our preliminary classifications. With these caveats in mind, below we describe the characteristics of the six motifs we judged to be strong riboswitch candidates (SRCs) on the basis of their structural characteristics and gene associations.

#### *The* pnuC *(SRC-10-1) motif*

Approximately 140 distinct representatives of a conserved motif were identified located almost exclusively upstream of and in the same orientation as *pnuC* genes in species within the genus *Streptococcus*. Published evidence indicates that some PnuC proteins transport nicotinamide riboside (NR) [41–43], which is a component of the ubiquitous coenzyme nicotinamide adenine dinucleotide (NAD*^+^*). Given that numerous riboswitch classes respond to nucleotide-based coenzymes [11], and that a recently reported riboswitch class was found to function with NAD^+^ [19,44], the *pnuC* motif RNA was immediately considered a promising ribo-switch candidate.

**Figure 1.**
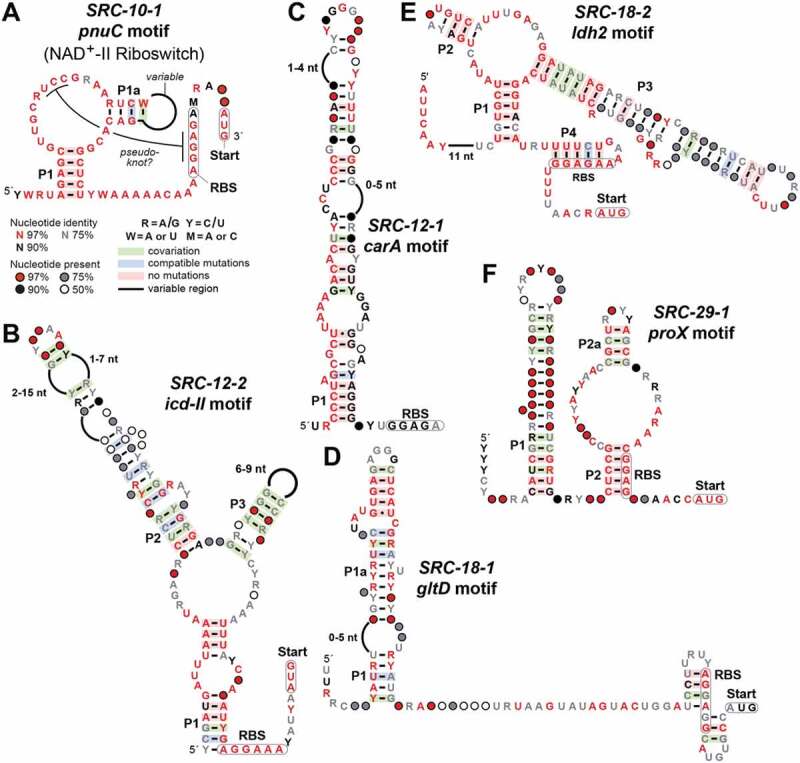
Consensus sequence and structural models for the (A) *pnuC* (NAD+-II riboswitch) [23], (B) *icd*-II, (C) *carA*, (D) *gltD*, (E) *ldh2* and (F) *proX* motif RNAs, which are among the collection of strong riboswitch candidates identified in this study. Annotations are as defined in the key depicted in A

Additional bioinformatic and biochemical analyses [23] identified a central core of the motif that is required for its function. These efforts trimmed the collection of non-redundant representatives to 43, which were used to create a consensus sequence and structural model (Fig. 1A). Each *pnuC* motif RNA appears to carry highly conserved nucleotides within an internal bulge formed by two stems. The RNA also has the potential to form a pseudoknot with the purine-rich ribosome binding site (RBS) [45] of the adjacent coding region, suggesting that binding of a ligand related to NAD^+^ metabolism might block ribosome binding to inhibit translation initiation [46]. This function would be analogous to that of a previously discovered riboswitch class for NAD^+^ [19]. These characteristics solidified our preliminary classification of the *pnuC* motif as a strong riboswitch candidate. Indeed, binding assays confirmed that *pnuC* motif RNAs selectively bind various nicotinamide derivatives, including NR and NAD^+^ [23]. Thus, each *pnuC* motif RNA serves as the aptamer for a second riboswitch class for this coenzyme, which we have named the NAD^+^-II riboswitch as published elsewhere [23].

#### *The* icd*-II (SRC-12-2) motif*

The *icd*-II motif (Fig. 1B) is represented by only 25 unique examples that are found mostly in the *Polynucleobacter* genus and metagenomic environmental sequences. Although originally found in *Polynucleobacter necessarius*, the same motif was also found through a search starting from one of the IGRs (SRC-28-2) of *Beta proteobacterium CB*, which has some degree of genetic similarity to members of the *Polynucleobacter* genus. This motif is located upstream of the *icd* gene, which codes for an NADP^+^-dependent isocitrate dehydrogenase (IDH) enzyme [47]. IDH is an important enzyme of the citric acid cycle, and thus it participates in managing the carbon flux through this energy metabolism pathway by supplying the cell with 2-oxoglutarate and NADPH [48]. The proposed secondary structure model consists of a three-stem junction [49], wherein the RBS for the adjacent ORF is predicted to participate in forming the first base-paired stem (pairing element 1, or ‘P1’). Thus, the *icd*-II motif appears to form an architecture that can regulate translation initiation of its associated ORF. However, the left shoulder of P1 is similar to a − 10 region of an RNA polymerase promoter sequence [50], and therefore we currently cannot be certain of the nucleotides that form the complete motif.

The *icd*-II motif is the second riboswitch candidate that has been discovered to associate with *icd* genes. A previously reported motif, called *icd* [35,51], is also found in species of Proteobacteria, although the representatives of these two motifs are present in different classes of this phylum. Both motifs are predicted to form a three-stem junction, but they differ both in their conserved sequences and the number of base-paired stems. Potential ligands for the *icd* motif are also potential ligands for the *icd*-II motif, such as citrate, oxaloacetate, glyoxylate, 2-oxoglutarate, or related compounds.

#### *The* carA *(SRC-12-1) motif*

The *carA* motif (Fig. 1C) has 30 unique representatives, each located immediately upstream of a *carA* gene. Again, although originally found in *Polynucleobacter necessarius*, this motif was also found in a search starting from one of the IGRs (SRC-28-1) of *Beta proteobacterium CB*. The gene *carA* codes for the small subunit of carbamoyl phosphate synthase, which is an enzyme that catalyzes the first committed step in pyrimidine and arginine biosynthesis [52]. The *carA* motif is found in β-proteobacteria, mostly in the *Polynucleobacter* genus, along with metagenomic environmental datasets. The proposed secondary structure consists of an extended imperfect hairpin, although the sparse representation provides little covariation support for this structure.

In *Escherichia coli*, the *carAB* operon is regulated by transcription factors binding to two promoters directly upstream of *carA* [53]. However, in genomes that contain the *carA* motif, there appears to be a promoter located upstream of this structure, but there does not appear to be a second promoter. Thus, it seems possible that this motif functions as a riboswitch, and that it substitutes for the function of a transcription factor system in organisms like *E. coli*. Notably, the conserved sequence and structural features are located immediately upstream of the predicted RBS and start codon for the adjoining ORF. This architecture is consistent with a possible *cis*-regulatory function where ligand binding regulates translation initiation. Carbamoyl phosphate or endpoints of biosynthetic pathways using this compound are potential ligands for this riboswitch candidate.

#### *The* gltD *(SRC-18-1) motif*

There are 95 distinct representatives of the *gltD* motif (Fig. 1D), although the majority of these are from bacterial DNA sequence information derived from metagenomic environmental samples that are not assigned to specific species. Representatives in sequenced organisms are exclusively derived from species of the Selenomonadales and the Veillonellales orders. This motif is frequently preceded by a predicted RNA polymerase promoter sequence, is always directly upstream of a predicted *gltD* gene, and is followed by a possible RNA hairpin structure that appears to occlude the RBS of the associated gene. All these features are consistent with an RNA motif that has a *cis*-regulatory function. The *gltD* gene encodes glutamate synthase which catalyzes the conversion of L-glutamine and 2-oxoglutarate into two molecules of L-glutamate [54]. A possible ligand for this riboswitch candidate might be related to either of the amino acids in this pathway. Intriguingly, a known riboswitch class for glutamine [55–57] is occasionally associated with glutamate synthase genes, but representatives of that previously discovered ribo-switch class are not present in the same species that carry the *gltD* motif. This lack of phylogenetic overlap strengthens the possibility that glutamine may be the ligand for the *gltD* riboswitch candidate.

#### *The* ldh2 *(SRC-18-2) motif*

The *ldh2* motif (Fig. 1E) has 34 unique representatives found in the *Negativicutes* class and various environmental DNA samples. It is almost exclusively found in front of genes associated with the *Ldh_2* conserved protein domain family whose representatives encode malate/L-lactate dehydrogenases [58]. The structure of this motif includes a three-stem junction containing highly conserved nucleotides and a long P3 stem with a central bulge that may cause it to kink back to form a binding pocket. A possible expression platform is present in the form of an RBS that may be hidden by the formation of an additional P4 stem. It is difficult to define a short-list of candidate ligands because of the uncertainty of the substrate and product of the associated gene product.

#### *The* proX *(SRC-29-1) motif*

The *proX* motif (Fig. 1F) has 17 unique representatives found primarily in the *Spiribacter* genus and in various environmental DNA samples from saltwater origin. It is found almost exclusively in front of the gene *proX*, which encodes a predicted glycine betaine/proline betaine transporter [59]. A highly conserved RBS that can be occluded by a corresponding sequence on the P2 stem provides a mechanism for plausible translational control. Given the importance of glycine betaine and proline betaine for osmoregulation of saltwater organisms, we speculate that this riboswitch candidate might respond to changes in the concentration shifts of one of these two molecules, or other osmoprotectants. Alternatively, the signalling molecule c-di-AMP is frequently used to signal osmotic distress [60–62], and a common riboswitch class for this compound has been reported previously [63].

### Weak riboswitch candidates

The following four riboswitch candidates described below are generally rare and may be associated with genes whose functions are not readily apparent. In some cases, we cannot provide a strong hypothesis for the possible metabolite or inorganic ion ligand that might be sensed by the candidate. Nevertheless, we judged them to be supported by sufficient structural and gene context evidence to classify them as weak riboswitch candidates (WRCs) and worthy of bringing to the attention of the research community. We advise those interested in pursuing the experimental validation of the motifs below to first pursue additional bioinformatics studies to strengthen their hypotheses.

#### *The* ilvD *(WRC-18-2) motif*

**Figure 2.**
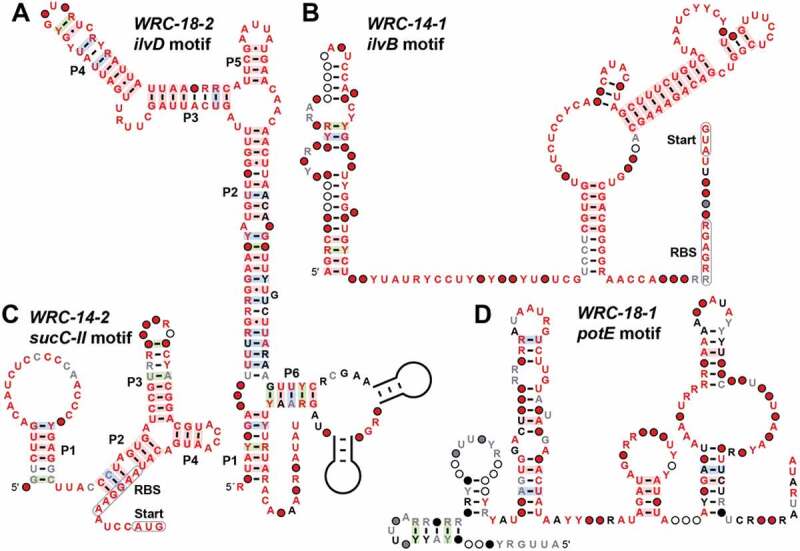
Consensus sequence and structural models for the (A) *ilvD*, (B) *ilvB*, (C) *sucC*, and (D) *potE* motif RNAs, which are among the collection of weak riboswitch candidates identified in this study. Annotations are as described for Fig. 1

There are 73 distinct representatives of the *ilvD* motif (Fig. 2A) found exclusively in the *Veillonella* genus of the Firmicutes phylum. The associated gene for all representatives is annotated as *ilvD*, which is predicted to encode a dihydroxyacid dehydratase enzyme that is a key contributor to the branched-chain amino acid biosynthesis pathway [64]. Some representatives have a potential intrinsic transcription terminator stem [65–67] (not depicted), which is positioned between the conserved, putative aptamer region and its associated ORF located downstream. This is a common characteristic of riboswitches that operate using an expression platform that permits ligand-mediated transcription termination [68,69].

Unfortunately, we cannot be certain that the *ilvD* motif functions as a riboswitch for several reasons. For example, the motif includes base-paired stems that are uncharacteristically long compared to most validated riboswitch classes. Furthermore, there is little evidence for covariation, which reduces our confidence in the proposed secondary structure. However, this latter weakness is not unexpected for a rare motif that lacks phylogenetic diversity among its representatives. In total, the characteristics of the *ilvD* motif suggest it likely acts as a cis-regulatory element, although it could serve as a small RNA (sRNA) [70,71] that regulates genes related to the biochemistry promoted by the *ilvD* gene product. We also cannot yet rule out the possibility that the *ilvD* motif functions as a protein-binding regulatory motif. However, we do not observe evidence typical of certain protein-binding nucleic acids, such as palindromic or short repetitive sequences.

Given the various points above, we have categorized the *ilvD* motif as a weak riboswitch candidate. Ligand candidates could be drawn from the list of compounds related to branched-chain amino acid biosynthesis pathways. This list also should include the bacterial signalling molecule ppGpp [72], which is sensed by a previously discovered riboswitch class that broadly controls branched-chain amino acid biosynthesis genes [14].

#### *The* ilvB *(WRC-14-1) motif*

There are only 7 unique representatives of the *ilvB* motif (Fig. 2B) and they are found exclusively in various species of the *Leptospira* genus. Thus, there is little evidence supporting the indicated conserved nucleotide positions, and there is little opportunity for nucleotides to exhibit covariation in stems. This motif is nevertheless notable because it is always found in front of the gene *ilvB*, which is another member of the branched-chain amino acid biosynthesis pathway discussed above for the *ilvD* motif. Similarly, the list of ligand candidates can be drawn from compounds related to branch-chain amino acid biosynthesis or the signalling molecule ppGpp.

#### *The* sucC*-II (WRC-14-2) motif*

There are only six representatives of the *sucC*-II motif (Fig. 2C) and they are also found exclusively in various species from the *Leptospira* genus. Despite the small number of hits, there is some evidence of covariation supporting the formation of the P1 and P3 stems. These motifs are exclusively found in front of the *sucC* gene which encodes the succinyl-CoA synthetase beta subunit [73]. This suggests a wide range of possible ligand candidates such as members of the citric acid cycle and a variety of secondary signalling molecules involved in maintaining cellular energy homoeostasis.

#### *The* potE *(WRC-18-1) motif*

There are 144 representatives of the *potE* motif (Fig. 2D) and they are found exclusively in members of the *Veillonella* genus and environmental sequence samples. They are almost always found in front of the gene *potE* which encodes a putrescine-ornithine antiporter [74]. Unfortunately, the motif’s secondary structure model is not well supported by covariation and it lacks an obvious expression platform. In addition, the motif is generally located some 200 nucleotides away from the downstream *potE* gene. However, the compelling gene association, possibly related to polyamine production [75], and the fact that a riboswitch for adenosylcobalamin (AdoCbl) [76] is almost always located in the IGR immediately downstream of *potE* causes us to consider this motif as weak riboswitch candidate.

### An unusual gene control element incorporating the odc1 (uORF-25-1) motif

**Figure 3.**
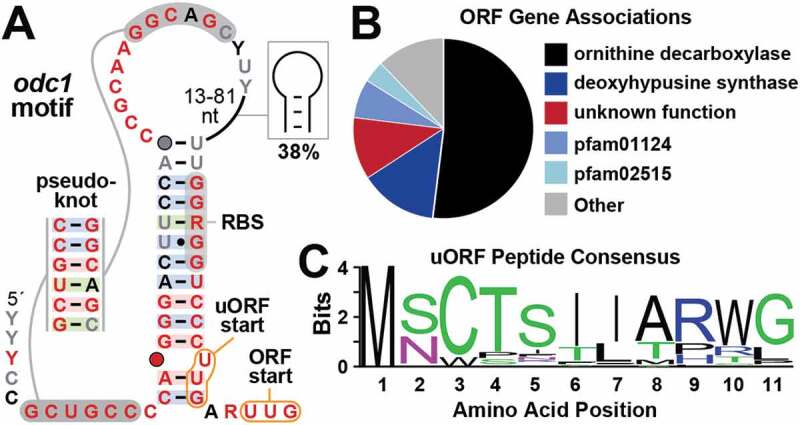
The *odc1* motif consensus model and gene associations. (A) Conserved nucleotide sequences and secondary structure model based on 272 representatives of *odc1* motif RNAs. (B) Distribution of gene associations for the *odc1* motif. Included are the first three genes downstream of the motif, which might constitute an operon. Genes annotated pfam01124 code for proteins of unknown function, and genes for pfam02515 code for putative CoA transferase enzymes also of uncertain function. (C) Consensus sequence (logo plot) for the peptide produced from the uORF start codon in all representatives of *odc1* motif RNAs

A particularly unusual gene control candidate called the *odc1* motif (Fig. 3A) was uncovered using the GC-IGR pipeline. This motif, which is well represented with 272 examples from species of the α-proteobacteria class, is predicted to form a hairpin with an extended stem structure (P1) closed by a relatively large loop. A pseudoknot appears to form between nucleotides near the 5ˊ terminus of the motif and nucleotides in the loop. The RNA domain is named for the most commonly annotated gene associated with the motif (Fig. 3B), which presumably codes for the enzyme ornithine decarboxylase. This enzyme converts ornithine into putrescine, which is the committed step to produce polyamines [77].

A predicted RBS forms part of the right shoulder of P1, and therefore is sequestered within the predominant secondary structure element depicted in the consensus model (Fig. 3A). Also included among the many highly conserved nucleotides that are characteristic of this motif is a predicted noncanonical UUG start codon for the main ORF. Intriguingly, another possible UUG start codon is always located immediately upstream of the main start codon, wherein only two nucleotides separate these two codons. Thus, the architecture of this motif indicates that the *odc1* motif likely has *cis*-regulatory function that controls the site of translation initiation.

Notably, the start codon for the upstream open reading frame (uORF) is predicted to initiate the synthesis of an 11 amino acid peptide wherein most amino acid positions are highly conserved (Fig. 3C). There is no evidence for variation of peptide length with any of the *odc1* motif representatives. These observations suggest that there is a functional role for this short peptide product. However, the well-conserved sequence and structural features of the complete *odc1* motif, largely encompassing nucleotides located upstream of the conserved uORF, suggests that the RNA might serve a larger role in choosing between the use of the two start codons during translation initiation. One possibility is that the motif acts as a metabolite-binding riboswitch. Perhaps, in the presence of a ligand, the RBS is sequestered by formation of the P1 stem to suppress expression from both start codons. In the absence of a ligand, an alternative structure might favour one start codon over the other, which would favour the production of either the short peptide from the uORF or the larger protein from the main ORF.

**Figure 4.**
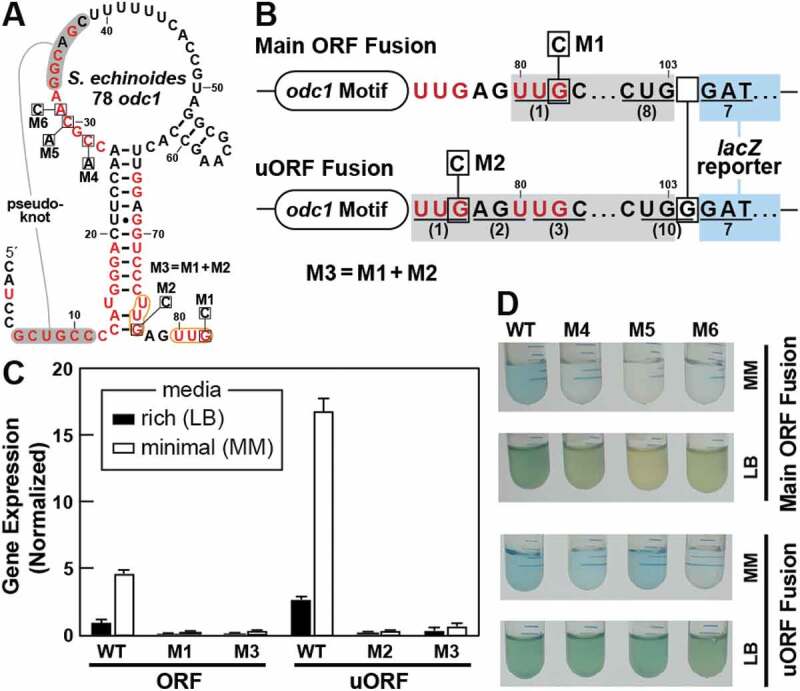
Expression of proteins from the two *odc1* translation start codons. (A) Sequence and predicted secondary structure of the wild-type *odc1* motif RNA from the *S. echinoides* representative. Red nucleotides identify conserved nucleotides of the *odc1* motif consensus as depicted in Fig. 3A. Nucleotide changes to create mutant constructs used in reporter gene assays are identified by boxed letters. Mutant construct M3 is the combination of mutations M1 and M2. (B) Reporter constructs were created by joining the *S. echinoides odc1* motif representative to an *E. coli lacZ* reporter gene. Top: Sequence of the construct fusing first 8 codons of the *odc1* gene (grey; codons underlined and numbered in parentheses) with the 7^th^ codon of the *lacZ* ORF (blue). Bottom: Sequence of the construct fusing the uORF start codon in frame with the *lacZ* ORF. Note that an insertion of a single G nucleotide after nucleotide 103 creates an in-frame fusion between the 10 codons of the *odc1* motif uORF and the 7^th^ codon of *lacZ*. Additional annotations are as described for A. (C) Plot of gene expression values for *E. coli* cells carrying various reporter fusion constructs as indicated and grown in media characterized as rich (LB) or minimal (MM). Values were normalized to those measured for cells carrying the WT main ORF construct and grown in rich medium. Bars represent the average of three replicates and the error bars indicate standard deviation. (D) Images of LB or MM liquid cultures supplemented with x-gal that were inoculated with *E. coli* cells carrying various reporter constructs as indicated

To investigate the general mechanistic features of the *odc1* motif, a reporter construct was created wherein the representative from the gram-positive bacterium *Sphingomonas echinoides* (Fig. 4A) was fused upstream of a β-galactosidase reporter gene. Two specific reporter fusion arrangements were prepared (Fig. 4B) to position the reporter gene either in the same reading frame as the main ORF by using the second start codon at positions 80 to 82, or in the same reading frame as the uORF by using the first start codon at nucleotide positions 75 to 77. In addition, certain mutant constructs (called M1 through M6, Fig. 4A) were made to examine the importance of highly conserved sequences and structural features of the *odc1* motif.

*E. coli* cells were used as a surrogate host organism to assess the function of the reporter constructs in vivo, which revealed several notable findings. For example, fusion of the reporter gene to either the main ORF or the uORF start codon of the wild-type (WT) *S. echinoides odc1* motif yields measurable but modest levels of expression (Fig. 4C). Expression from the uORF start codon is approximately 3-fold greater than that observed for the main ORF start codon, regardless of whether rich or minimal medium is used. Notably, both ORFs exhibit higher gene expression levels in minimal media (M9) than in rich media (LB). The modest amount of gene expression from either reporter construct is largely eliminated when a disruptive mutation is made to its corresponding start codon (M1 through M3, Fig. 4C), which confirms that protein synthesis is being driven by the predicted sites for translation initiation. Importantly, these results reveal that both start codons can be utilized by the mRNA, indicating that the proposed 11 amino acid peptide (Fig. 4C) is produced from the start codon associated with the uORF.

Next, we examined whether the motif serves as a genetic control element and whether highly conserved nucleotides near the pseudoknot are important for this function. Mutant constructs M4 through M6 were prepared that carry changes to strictly conserved positions immediately preceding the nucleotides predicted to form part of the pseudoknot (Fig. 4A). All three mutations caused a loss of expression of the reporter gene when fused in-frame with the main ORF start codon. In contrast, mutations present in M4 and M5 had little effect on reporter gene expression when initiated by the start codon of the uORF. Again, although the level of gene expression is modest, these results suggest that certain conserved nucleotides in the *odc1* motif are important for regulating translation, particularly for the main ORF. However, additional experiments will be needed to determine how these nucleotides participate in affecting the level of gene expression from the adjacent start codons.

### Additional notable motifs identified with the GC-IGR pipeline

The GC-IGR bioinformatics pipeline yielded numerous additional motifs that we have not included on our list of candidate riboswitch classes. Some motifs are rare, and thus lack sufficient clues for us to responsibly speculate on their possible functions. However, some candidates have properties that are suggestive of functions that are different from gene control via riboswitch action. Several of the most intriguing candidates are briefly described below.

#### The PBC-28-3 motif

**Figure 5.**
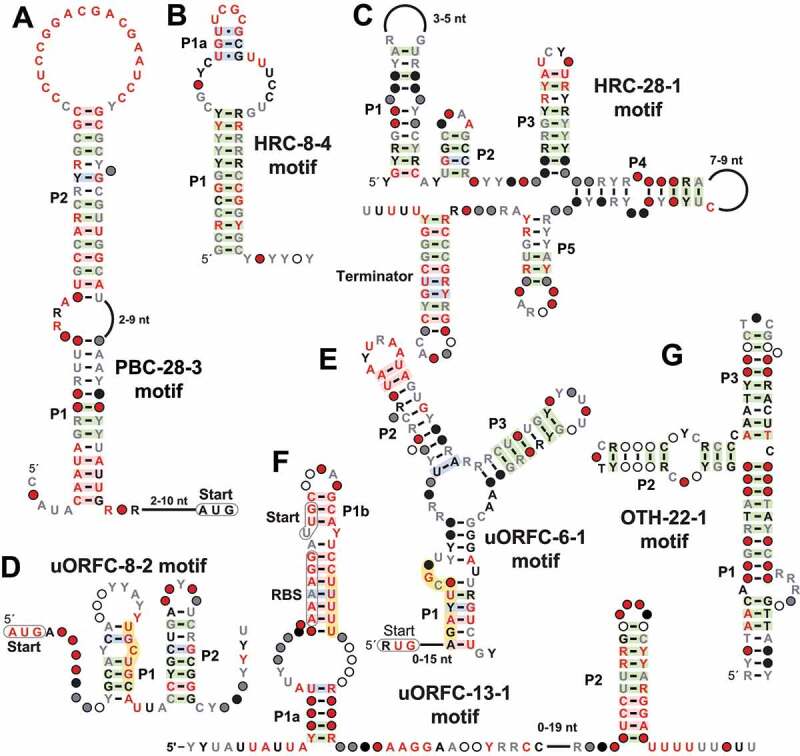
Consensus sequence and secondary structure models for additional structured nucleic acid motifs that are representative of those identified in this study. Depicted is (A) a putative protein-binding candidate PBC-28-3, two high-ranking candidates (B) HRC-8-4 and (C) HRC-28-1, three uORF candidates (D) uORFC-8-2, (E) uORFC-6-1, and (F) uORFC-13-1, and (G) a predicted structured DNA motif OTH-22-1

A total of 21 unique examples of the PBC-28-3 motif (Fig. 5A) are found upstream of genes that encode proteins closely related to AhpC, a peroxiredoxin, which forms an important component of the bacterial defence system against toxic peroxides [78]. The motif appears to be cis-regulatory because it is almost always oriented in the same direction as the downstream gene. Also, the start codon for the adjacent ORF is located immediately next to the motif, suggesting that regulation of translation might be its function. The PBC-28-3 motif RNA consists of a long covarying stem with a highly conserved loop. This loop includes regions of pyrimidine nucleotides, which is suggestive of a possible role in associating with protein factors that prefer polypyrimidine binding sites. This characteristic, in addition to the long base-paired substructures, suggests this RNA might function as a protein binding motif rather than as a riboswitch that binds a small molecule ligand.

#### The HRC-8-4 motif

The consensus sequence and secondary structure model of the HRC-8-4 motif (Fig. 5B) was determined from 160 unique representatives exclusively uncovered from species of the Rhodobacterales order of the α-proteobacteria class. The motif is primarily located in between the *ccoNOQP* and *ccoGHIS* operons that both code for genes necessary for the maturation of cbb3-type cytochrome c oxidase complex [79]. Usually the *ccoP* gene is found immediately upstream and the *ccoG* gene immediately downstream, but in some instances there are other genes adjacent to the motif between the two cytochrome c oxidase operons. The most commonly inserted gene is *hvrB* that encodes a LysR-family transcriptional regulator [80]. When the *hvrB* gene is present, it is always located immediately downstream of the HRC-8-4 motif. There are also some arrangements where the motif is upstream of the *hvrB* gene when it is not adjacent to cytochrome c operons.

Structurally, the HRC-8-4 motif vaguely resembles the recently validated HMP-PP [17] and guanidine-IV [21,22] riboswitch classes. Like these experimentally validated ribo-switch classes, the HRC-8-4 motif generally conforms to the classic architecture of an intrinsic terminator stem [64–66], which includes a strong stem followed by a run of U nucleotides. However, they also carry well-conserved nucleotides at the tip of the stem-loop structure, which is unusual for terminator stems. HMP-PP and guanidine-IV riboswitches appear to exploit these conserved nucleotides to form the aptamers for their target ligands. This precludes the formation of the terminator stem when ligands bind and thus they function as genetic ‘ON’ switches [17,21,22]. We speculate that the HRC-8-4 motif might perform a similar function, although we again cannot be certain that the ligand is a small molecule. The frequent association of the *hvrB* gene might indicate that this nucleic acid binding protein might bind to this terminator stem to regulate its own expression.

#### The HRC-28-1 motif

The HRC-28-1 motif (Fig. 5C) is represented by 33 unique examples mostly found in species of *Polynucleobacter*. The most commonly associated upstream gene encodes a provisionally annotated oxidative damage protection protein that is a member of the CDD family PRK05408 [81]. The most commonly associated downstream gene is the ribose-5-phosphate isomerase *rpiA* [82], which is always oriented in the opposite direction as the HRC-28-1 motif. The proposed secondary structure model for the motif includes six stems, where the final base-paired substructure appears to be an intrinsic terminator. The motif is unlikely to be a riboswitch due to its orientation relative to adjacent genes, but rather may be a structured RNA element located in the 3ˊ-UTR of the mRNA encoded by the upstream gene.

#### The uORFC-8-2 motif

There are 145 unique examples of the uORFC-8-2 motif (Fig. 5D) that are found exclusively upstream of the *cysS* gene of the Rhodobacterales order in the α-proteobacteria class. The *cysS* gene encodes a cysteinyl-tRNA synthetase, and thus it is not surprising that this motif has characteristics expected for a ribosome-mediated attenuation sequence [83]. Specifically, we note the presence of a conserved AUG start codon for a uORF, wherein two highly conserved cysteine codons appear in tandem usually at positions 14 and 15 of the resulting short polypeptide (Fig. 5D, orange shading). The UGC codon for cysteine dominates, but there are examples of the UGU codon as well.

Structurally, the motif forms two prominent stems with the first containing the cysteine codons. In some cases, there is the possibility that a third base-paired region can form to create a three-stem junction. However, these cases are rare and lack evidence for covariation. In all cases, the consensus model includes a run of U nucleotides at the 3ˊ terminus, which is consistent with the formation of an intrinsic terminator stem. Presumably, ribosome stalling at the cysteine codons due to inadequate levels of aminoacylated tRNA^Cys^ permits transcriptional read-through and production of the mRNA encoding the CysS protein, which generates more cysteinyl-tRNA^Cys^.

#### The uORFC-6-1 motif

The uORFC-6-1 motif (Fig. 5E) is a uORF candidate with 51 unique representatives predominantly found in the Bacillales order of Firmicutes. It is primarily found upstream of the gene *hflX*, which encodes an RNA helicase involved in rescuing stalled and heat-damaged ribosomes [84]. The RNA secondary structure of this motif is entirely located downstream of the predicted start codon for the uORF and is supported by strong evidence of covariation. In addition, the peptide sequence has a conserved Arg-Leu-Arg motif (Fig. 5E, orange shading), which has been found previously in antibiotic resistance leader peptides that rely on antibiotic-induced ribosome stalling to turn on the expression of downstream genes [85]. The presence of this ORF within the predicted RNA structure suggests a similar mechanism whereby the presence of stalled ribosomes on the transcript at the uORFC-6-1 motif might influence transcription of the downstream main ORF coding for the HflX protein.

#### The uORFC-13-1 motif

The uORFC-13-1 motif is found only in species from the genus *Acholeplasma* and from environmental sequences where the organisms are unknown. There are 24 unique sequence representatives, which are located exclusively upstream of the *aroF* gene encoding the enzyme 3-deoxy-7-phosphoheptulonate synthase. This enzyme produces the first compound in the shikimate pathway that leads into the biosynthesis of the amino acids phenylalanine, tyrosine, and tryptophan. The motif is located immediately upstream of an apparent intrinsic transcription terminator stem, suggesting this motif regulates transcription termination. Features of the consensus model (Fig. 5F) reflect its likely function as a uORF, including possible −10 and −35 promoter regions, an RBS and start codon, and a run of U nucleotides that code for at least one phenylalanine residue. These features suggest that gene regulation might involve the speed of phenylalanine incorporation into a peptide encoded by the uORF, in a process that might control the formation of the adjacent terminator stem. Comparative sequence analysis also supports the formation of another base-paired substructure, and therefore the motif might have additional functional features. A similar attenuation-based mechanism for the control of *aroF* in *E.coli* has been previously hypothesized [86], although the uORFC-13-1 motif differs from the 5′ UTR found in that organism.

#### The OTH-22-1 motif

The OTH-22-1 motif (Fig. 5G) has 1887 unique representatives found in several bacterial phyla as well as in Archaea and environmental sequences. The RYYYAAC consensus sequence, found at the 5ˊ terminus of the motif is also a distinguishing characteristic of *attC* structured DNA elements [87]. Because this motif contains the same consensus sequence with comparable secondary structure, this motif is likely another variant of this type of structured DNA element. However, none of the representatives of this motif overlap with annotations of known classes of *attC* sites, indicating that this is likely a novel form of these single-stranded DNA motifs.

### Comparison of bioinformatic and transcriptomic search results for *Listeria monocytogenes* RNA regulatory motifs

The inclusion of *L. monocytogenes* among the collection of 26 genomes analysed in this study provided the opportunity to contrast the results from our computational approach (GC-IGR search) [38] with the transcriptomics-based experimental approach (term-seq) [88]. The term-seq approach generates sequencing reads on a genome-wide scale that establishes the natural 3ˊ termini of RNA transcripts. This data can then be examined to detect signatures of regulatory RNAs such as riboswitches. For example, a riboswitch that terminates transcription immediately upstream of its associated mRNA ORF region will exhibit a higher abundance of sequencing reads spanning its aptamer region compared to those for the downstream ORF. Indeed, term-seq data yielded such signatures for 28 previously known riboregulators in *L. monocytogenes*, including 13 riboswitches, 12 T-box leader sequences, and three protein-binding leader sequences. Also, the authors concluded that 12 additional novel regulatory RNA regions are detected by the term-seq approach, although 10 of these 12 novel regulatory regions had been previously identified as expressed sRNAs [89–91].

Interestingly, none of the 12 proposed novel regulatory RNAs from term-seq passed through our initial IGR filtering process. Specifically, the IGRs corresponding to eight of the riboregulator candidates were excluded from analysis because they were already annotated in Rfam [92], and thus are not considered unknown IGRs. The remaining four, including two previously identified sRNAs whose annotations were not present in Rfam, were excluded from the filtering stages of our computational pipeline because of insufficient IGR length or GC content. In other words, they do not exhibit the characteristics typical of most other IGRs that contain structured bacterial ncRNAs such as riboswitches.

To further assess these 12 term-seq candidates, we subjected them to comparative sequence analysis in search of conserved nucleotide sequences or secondary structure features. A more detailed description of each of these motifs is included in the supplementary information (Figs. S33-S34). None of the 12 IGRs exhibited the combination of conserved nucleotides, structural complexity, and a genomic context theme that are typical of strong riboswitch candidates or other RNAs that rely on sophisticated secondary or tertiary structures for their function. Rather, the IGRs mostly appear to carry an intrinsic terminator stem [65–67] without evidence for complex structure formation beyond this genetic element. These RNAs might carry the regulatory equivalent of a riboswitch expression platform, but they appear to lack the accompanying aptamer domain that is needed to selectively bind the target ligand to trigger changes in gene expression.

### Summary of the motifs discovered and implications for future searches

**Figure 6.**
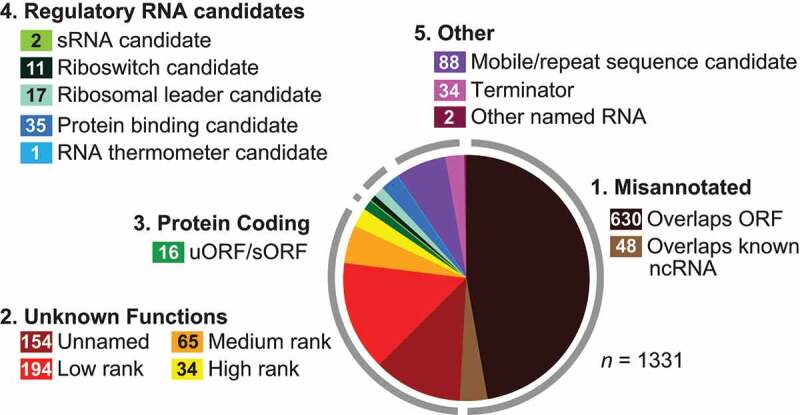
Comprehensive summary of classification of selected unknown IGRs (1331 total) from the analysis of 26 bacterial genomes chosen for this study. IGR classifications are placed into five groups. (1) Originally annotated IGRs that either code for ordinary-length proteins or serve as templates for known types of ncRNAs. (2) IGRs that are judged to have varying degrees of promise as structured ncRNAs but lack sufficient evidence to assign a possible function. (3) Originally annotated as IGRs but are now predicted to code for short peptides. (4) IGRs that likely serve as templates for the transcription of ncRNAs, including regulatory RNA candidates and selfish (mobile/repeat) sequences. (5) Transcription terminator structures or other sequences. The numbers of candidates in each sub-category are provided in the coloured boxes

The GC-IGR analysis of 26 bacterial genomes has led to the discovery of a diverse collection of novel structured ncRNA motifs and other genetic elements (Fig. 6). Approximately half of all IGRs analysed in detail were found to be previously misannotated, and appear to serve either as coding regions for known proteins or as transcription templates for known classes of ncRNAs. This extent of misannotation is similar to that observed with our previous effort to examine five bacterial genomes [38]. Perhaps as automated annotation algorithms improve, these inappropriately classified IGRs will be reduced in number, which would help accelerate genome analysis using the GC-IGR pipeline.

Another finding that is consistent with our previous study is that a large portion of IGRs that cluster with those carrying known ncRNAs do not exhibit sequence or structural homology with other IGRs, sometimes even from closely related species. Perhaps additional representatives for these rare IGR types will be found as the bacterial genomic DNA sequence databases expand. However, some of these IGRs could represent exceedingly rare ncRNA classes, which are predicted to exist based on the distribution of abundances for known riboswitch classes [11]. Alternatively, some of these IGRs might have no function derived from their sequences and structures, and thus truly represent junk DNA sequences that have no value to the cell.

Despite the large number of misannotated and unassigned IGR functions, a variety of novel regulatory RNA candidates were uncovered by the GC-IGR pipeline. Approximately 5% of all analysed IGRs are predicted to carry regulatory RNAs. Of particular interest to us are riboswitches, and this study has revealed the existence of at least 10 reasonable candidates (Fig. 1 and Fig. 2). Indeed, one of these, initially called the *pnuC* motif (Fig. 1A), has proven to function as a riboswitch for NAD^+^ [23]. Many additional candidates also have characteristics suggestive of gene control functions, but additional experiments will be necessary to establish the true functions of these conserved ncRNA structures. However, if this collection of riboswitch candidates yields several validated riboswitch classes, these findings would be consistent with our prediction that many more regulatory RNAs such as riboswitches remain hidden among the sequenced bacterial genomes [11,30,31]. Given that many thousands of bacterial genomes have the properties needed for successful application of the GC-IGR pipeline, we believe that many hundreds of classes of structured ncRNA candidates could be uncovered just among the bacterial genomes that have been sequenced to date.

## Concluding remarks

In the current study, we have expanded the number of phyla represented by the species subjected to the GC-IGR search pipeline to nine, from the three phyla sampled in our previous study [38]. In addition to the new RNA discoveries, our findings again demonstrate the utility of the GC-IGR pipeline. This computational system offers a means to efficiently identify nearly all ncRNA motifs in a given genome by examining unknown IGRs that cluster near known ncRNA representatives based on length and GC content characteristics. The GC-IGR analysis can be applied to multiple different genomes without the need for experimental manipulation, which permits the large-scale analysis of many different genomes.

Indeed, the GC-IGR pipeline has advantages over transcriptomics-based methods (e.g [88].) in several ways. For example, a bioinformatics approach only requires a sequenced genome and does not require the ability to culture the species of interest to isolate and prepare RNA transcripts for subsequent analysis. In addition, the GC-IGR method can identify structured ncRNA motifs regardless of whether they are transcribed only under certain growth conditions. However, this method works best for identifying ncRNA candidates in organisms whose genomes are strongly biased either against or in favour of G and C nucleotides. These extreme biases cause IGRs carrying templates for structured ncRNAs to cluster apart from most other IGRs from these genomes in plots of %GC content versus IGR length.

The findings from the 26 genomes reported in this study, along with the results from the analysis of five genomes previously [38], demonstrate that a great diversity of novel ncRNA motifs, and even some structured single-stranded DNA elements, can be discovered by applying a more exhaustive bioinformatic analysis of sequenced bacterial genomes. Through the current study, along with our pilot applications of the GC-IGR pipeline [37,38], we conclude that it is possible to scale-up the application of the search method. However, certain barriers must be overcome to discover candidate regulatory RNAs and other ncRNA motifs that are of most interest to RNA or microbiology researchers. Over half of the IGRs that were analysed in the current project eventually could be ruled out as novel structured ncRNA candidates (Fig. 6). Substantial time is required to manually improve the genome sequence annotations to remove IGRs that code for known proteins or that function as templates for the transcription of known ncRNAs. Improvements to the pipeline that reduce the required manual contributions to the search process will speed the analytical process. However, it is already practical to apply the GC-IGR pipeline to hundreds or even thousands of bacterial genomes. If implemented, such searches would undoubtedly yield many novel ncRNA classes.

Although we cannot precisely define the size of the undiscovered pool of ncRNAs in the bacterial domain of life, this collection must be vast given the great number and diversity of bacterial species on the planet, along with their propensity to use structured ncRNAs to achieve various tasks. Hundreds of additional riboswitch classes are predicted [11,30,31] to await discovery and validation among the bacterial genomes whose sequences are already available, although the vast majority of these classes are expected to be rare and therefore only narrowly distributed. Rarer riboswitch classes will pose challenges for discovery by search strategies that rely on comparative sequence analysis algorithms. However, the abundance of sequenced bacterial genomes coupled with the application of search strategies such as the GC-IGR pipeline should continue to yield numerous candidate riboswitch classes and many other structured ncRNA motifs. Thus, despite the conclusion that numerous structured ncRNA classes remain to be discovered, the rarity of each individual motif means that the genome of each bacterial species is likely to carry very few if any novel ncRNA classes.

The genomes that produced the most riboswitch candidates when subjected to the GC-IGR analysis pipeline belong to the two α-proteobacterial species from among the 26 species that were analysed in the current study. Notably, Proteobacteria constitute the phylum with the second most abundant representation of riboswitches [11]. Thus, our GC-IGR pipeline is uncovering rare and obscure riboswitch candidates primarily in organisms from bacterial domains that previously yielded the most candidates. This suggests that future riboswitch discoveries will likely be concentrated in species from these riboswitch-favouring lineages.

As noted above, our findings also highlight the growing difficulty for those who seek to discover novel riboswitch classes. Distinct riboswitch candidates account for only about 1% of all analysed IGRs, even after the IGRs are sorted based on IGR length and GC content to favour the analysis of those with characteristics most similar to known riboswitch classes. At the current pace, we are encountering an average of one new riboswitch candidate for every two to three bacterial genomes analysed based on our current findings, previous publications [37,38], and unpublished observations. This ratio likely could be improved by biasing our searches towards genomes from bacterial lineages that are known to be enriched for riboswitches. However, as novel riboswitch candidates are uncovered, it is expected that this ratio will continue to decline as the more common and noticeable classes are identified.

The critical problem is that the remaining hidden classes should trend towards the rare and the structurally obscure. As the number of natural homologs decreases, so does the ability to detect the candidate motif. We believe that by pre-sorting IGRs for desirable length and %GC composition, we improve the chances of uncovering rare but biologically relevant ncRNA motifs that might be supported by limited comparative sequence analysis data. Without pre-sorting, rare ncRNA motifs might be lost in the noise of false positives generated by comparative sequence analysis using all IGRs. Even if a novel motif is identified, fewer distinct representatives should reduce the ability to establish a quality consensus sequence and structural model and reduced confidence in the tentative classification of the candidate.

Unfortunately, the research community has not yet created an approach that could comprehensively uncover novel riboswitches on a large scale without employing a massive parallel experimental testing approach (which would be impractical as further noted below). One future possibility would be to use a structural modelling system that predicts 3D structures of all possible ncRNAs in a bacterial genome based on only one sequence. This capability does not yet exist, and even if it did, riboswitch aptamers that require ligand docking to adopt their stable aptamer structure are unlikely be recognizable as part of a riboswitch. Thus, comparative sequence analysis, even with sparsely represented ncRNA classes might be required to discover novel riboswitch classes in large numbers for the foreseeable future. Even with this substantial and growing technical burden, a large number of novel regulatory RNAs are accessible through the use of the GC-IGR pipeline. Although we are working to implement new computational tools to accelerate the GC-IGR pipeline, it is already possible to use the current pipeline to systematically analyse all the suitable bacterial genomes available to uncover hundreds of reasonable riboswitch candidates.

Given the capability of this search strategy to yield many additional riboswitch candidates, and the abundance of existing orphan riboswitch candidates [51], it seems appropriate to question the academic value and practical utility of discovering and validating more classes. However, we anticipate that numerous surprising findings await the discovery of additional riboswitch classes. Some of these novel motifs are rare and/or exceedingly simple in architecture but might perform sophisticated ligand sensing and gene control functions. For example, a notable riboswitch candidate uncovered by our recent implementation of the GC-IGR pipeline is the ‘*thiS*’ motif [38]. This simple hairpin structure has proven to function as a riboswitch for the thiamin pyrophosphate precursor called HMP-PP [17]. Experimental validation of this unusual ncRNA candidate has demonstrated a unique riboswitch regulatory mechanism as well as revealed another form of biochemical control of the thiamin pyrophosphate biosynthesis pathway. Given the architectural similarity between HMP-PP riboswitches and the HRC-8-4 motif uncovered in the current study (Fig. 5B), we are optimistic that additional riboswitches exist that make use of very simple terminator-embedded aptamers to control gene expression. If true, the GC-IGR pipeline is particularly well suited to uncover additional versions that exploit this riboswitch mechanism.

Furthermore, numerous other types of structured ncRNA and ncDNA elements are uncovered by implementing the GC-IGR pipeline (Fig. 5). Many of these will be readily assigned to a few well-understood classes of RNA or DNA genetic elements. However, others will have unusual characteristics, or be modestly represented, such that predicting their functions without additional information will be problematic. Currently, we bin these motifs into high-, medium- and low-ranking candidates, although future experiments or additional bioinformatics information might allow them to be grouped into a known class. However, some of these undefined motifs will likely represent entirely new classes of nucleic acids that carry out novel functions.

Despite our arguments supporting the projection that thousands of riboswitch classes remain to be discovered [11,30,31], it is unrealistic to expect that any single bacterial species will have a dozen or more novel classes of metabolite-responsive riboswitches. The huge number of riboswitch classes proposed to remain undiscovered can be reached even if each bacterial species has an average of less than one novel riboswitch class. Therefore, transcriptomics methods such as term-seq [88] are unlikely to provide a practical means to uncover these hidden classes more broadly among bacterial species. Specifically, it is not possible to culture all bacterial species in a laboratory setting, and even cultured species might need to be grown under many different conditions to generate a transcriptomics pattern that reveals the presence of a rare riboswitch class. In contrast, DNA sequences can be obtained for all bacterial species whose genome can be sampled, and bioinformatics methods such as the GC-IGR search pipeline can reveal relatively rare riboswitch classes.

It is important to note that the term-seq approach can uncover metabolite-binding riboswitch representatives, as was demonstrated using this approach to rediscover 13 previously known examples [88]. However, given the relative rarity of each undiscovered riboswitch class, and the low likelihood of finding a novel riboswitch class in any specific bacterial genome, experimental methods such as term-seq will produce a far greater collection of short RNA transcripts that have functions distinct from metabolite-binding riboswitches. As we have noted previously [38], the GC-IGR search strategy is not well suited to uncover simpler RNA motifs such as are characteristic of sRNAs [71] or regions that code for short peptides. This, coupled with the enrichment of candidate IGRs by length and GC content, provides a more effective means to uncover novel riboswitch classes from a large number of bacterial species.

## Materials and methods

### Databases and bioinformatics

Bacterial genome sequences from Reference Sequence (RefSeq) [93] database (release 76) and metagenomic datasets previously described [94] were used for the initial searches. Methods for IGR selection and analysis are described in detail elsewhere [38]. Briefly, a Matlab script was used to label IGRs and create plots based on %GC and length. The script allows a user to draw a selection polygon over the region that is enriched for known structured ncRNAs, which we usually limited to a region including approximately equal numbers of unknown IGRs and known ncRNAs.

Selected unknown IGRs were used as a basis for a BLASTX [95] search and IGRs with substantial overlap with proteins in NCBI’s nr database are marked ‘Known ORF’. Each of the remaining IGRs are searched using Infernal 1.1 [96] against a database of microbial IGRs (RefSeq 80) [97] and a database of metagenomic DNA sequences [36]. The resulting motifs with substantial overlap with known Rfam families were labelled ‘Known RNA’. Motifs with three or fewer representatives are labelled ‘Unnamed IGR’.

The remaining IGRs were refined by an iterative search process that uses CMfinder [98] to generate possible covariation models and Infernal 1.1 to find additional representatives that fit revised models. CMfinder yields RNA structural alignments that were used to determine if the motif carries additional or alternative structures not previously identified. Consensus sequence and structural models were generated with the program R2R [99]. The first gene downstream of each representative of a motif was used to generate genetic context graphics, as this is the gene most likely controlled by a *cis*-regulatory element. If no genetic information is available for a representative, it was excluded from the genetic context data. After one or more cycles of refinement, motifs were assigned a hypothesized function based on the consensus models and predominant nearby gene associations. Motifs with uninformative nearby gene contexts were assigned the categories ‘High-, Medium-, and Low-ranking candidates’ based on the number of hits and our relative confidence in the proposed consensus model as described previously [38].

### Chemical and oligonucleotides

All chemicals and chemically synthesized oligonucleotides were purchased from Sigma-Aldrich. Enzymes were purchased from New England BioLabs unless otherwise noted.

### Bacterial strains and growth conditions

*E. coli* BW25113 was obtained from the Coli Genetic Stock Center (Yale University). Reporter vector pRS414 was a gift from W. W. Simons (UCLA). Cells were grown in Lysogeny Broth (LB) or in M9 broth (1X M9 salts [42 mM Na_2_HPO_4_, 24 mM KH_2_PO_4_, 9 mM NaCl, 19 mM NH_4_Cl], 1 mM MgSO_4_, 0.1 mM CaCl_2_, 2% glucose, 0.5 µg/mL thiamin) purchased from Teknova. When required, growth medium was supplemented with carbenicillin (100 μg/mL).

### Reporter gene construct design

Sequences of DNA primers used for cloning are included in **Table S2**. In-frame plasmid reporter fusion constructs were created via PCR of a DNA fragment that contains the *B. subtilis lysC* promoter and the region encompassing the *odc1* riboswitch candidate from *S. echinoides* ATCC 14,820, extending through the first 8 codons of the main ORF. This DNA segment was cloned into the translational reporter vector pRS414, where the 8^th^ codon of the main ORF associated with *odc1* was fused with the 7^th^ codon of the *lacZ* reporter gene. The analogous uORF plasmid reporter fusion construct was created by including an additional G nucleotide between nucleotides 103 and 104 of the natural sequence (Fig. 3B). The resulting plasmid was transformed into *E. coli* BW25113. Mutant reporter strains were prepared from these two parent constructs using synthetic primers containing the relevant mutations.

### Liquid-based β-galactosidase assays

Reporter gene assays were conducted as previously described [95]. For liquid-based β-galactosidase assays, a single colony of the relevant *E. coli* reporter strain was picked and grown overnight in LB medium supplemented with carbenicillin. Cells were then washed twice with phosphate buffered saline (PBS), then diluted 1:200 in either LB or M9 minimal medium and incubated for 6 h at 37°C in various growth conditions. Visual detection of reporter gene expression was achieved by supplementing liquid media with X-gal (50 μg mL^−1^). To measure reporter gene expression, 80 μL of each resulting culture was added to a black Co-Star 96-well clear-bottom assay plate and the absorbance at 595 nm was measured using a Tecan Synergy 2 plate reader. Cells in each well were then mixed with 80 μL of Z buffer (60 mM Na_2_HPO_4_, 40 mM NaH_2_PO_4_, 10 mM KCl, 1 mM MgSO_4_), after which 40 μL of 4-methylumbelliferyl-β-D-galactopyranoside (40 µl of a 1 mg mL^−1^ solution) was added and mixed thoroughly. Plates were incubated at room temperature for 15 min, and the reaction was quenched by the addition of 40 µL of 1 M Na_2_CO_3_ solution. Excitation and emission were measured at 360/460 nm using a Tecan Synergy 2 plate reader, and fluorescence units were calculated as previously described [16,100].

## Supplementary Material

Supplemental Material
