# Supplementary material for: Comprehensive discovery of novel structured noncoding RNAs in 26 bacterial genomes
Source: RNA Biol. 2021 May 10;18(12):2417–32. doi: 10.1080/15476286.2021.1917891 (PMC8632094; doi:10.1080/15476286.2021.1917891)
Supplement: Supplemental Material [file KRNB_A_1917891_SM4594.docx]

***Supplementary Information***

**Comprehensive discovery of novel structured noncoding RNAs in 25 bacterial genomes**

**Kenneth I. Brewer^a,*^, Etienne B. Greenlee^b,*^, Gadareth Higgs^b,*^, Diane Yu^b,*^, Gayan Mirihana Arachchilage^c^, Xi Chen^a^, Nicholas King^a^, Neil White^c^, and Ronald R. Breaker ^a,b,c^**

^a^Department of Molecular Biophysics and Biochemistry, ^b^Department of Molecular, Cellular and Developmental Biology, ^c^Howard Hughes Medical Institute, Yale University, P.O. Box 208103, New Haven, CT 06520-8103, USA.

*These authors contributed equally to the project.

Contact: Ronald R. Breaker

Email: [ronald.breaker@yale.edu](mailto:ronald.breaker@yale.edu)

Phone: 203 432-9389

**Plots of all 26 analyzed genomes**

The IGRs of the genomes chosen for analysis are plotted by their IGR length and their %GC. A summary of all analyzed genomes is listed in **Supplementary Table 1** and the corresponding plots for each genome can be found in **Supplementary Figures S1 – S26**.

**Supplemental Table S1.** The list of all 26 genomes included in this analysis. The genome numbers (Gen. #) for this series begins at 6 because this analysis extends the data derived from the five genomes analyzed previously (Stav S, et al., BMC Microbiol 2019; 19:66.). Acc. # is the accession number of the species. Gen. %GC represents the GC content of the organism’s genomic DNA. “IGR %GC” is the GC content of all IGRs in the genome, and “IGR Length” is the average length of all IGRs in the genome. “Total IGRs” represent the number of IGRs in the entire genome. Examined IGRs represent the number of IGRs selected for detailed analysis. The %GC versus IGR length plots for genomes 6 through 31 correspond to **Supplementary Figures S1** through **S26**, respectively.

**Gen. IGR IGR Total Examined**

**Gen. # Acc. # Organism %GC %GC Length IGRs IGRs**

6 NC_003210.1 *Listeria monocytogenes* 38.0 34 131 2439 94

7 NC_003454.1 *Fusobacterium nucleatum*  27.0 23 155 1679 98

8 NC_008044.1 *Ruegeria* sp. TM1040 60.1 59 152 2450 74

9 NC_008261.1 *Clostridium perfringens* ATCC 13124 28.4 21 241 2142 60

10 NC_008525.1 *Pediococcus pentosaceus*  37.4 30 189 1072 29

11 NC_008599.1 *Campylobacter fetus* subsp. *fetus* 82-40 33.3 24 138 1090 44

12 NC_009379.1 *Polynucleobacter necessarius*  44.8 38 84 1769 64

13 NC_010163.1 *Acholeplasma laidlawii* PG-8A 31.9 27 125 1039 61

14 NC_010602.1 *Leptospira biflexa*  39.1 36 100 2501 142

15 NC_011653.1 *Thermosipho africanus*  30.8 31 129 1338 36

16 NC_012673.1 *Exiguobacterium* sp. AT1b 48.5 41 157 1830 44

17 NC_012968.1 *Methylotenera mobilis*  45.5 37 119 1994 16

18 NC_013520.1 *Veillonella paravula*  38.6 32 176 1576 74

19 NC_014109.1 *Candidatus Riesia pediculicola* USDA 28.6 21 228 407 25

20 NC_014166.1 *Arcobacter nitrofigilis* DSM 7299 28.4 22 103 2275 51

21 NC_014921.1 *Mycoplasma fermentans* M64 26.9 21 155 681 25

22 NC_015318.1 *Hippea maritima*  37.5 37 106 968 32

23 NC_015682.1 *Thermodesulfobacterium geofontis*  30.6 24 90 1147 28

24 NC_016043.1 *Taylorella asinigenitalis* MCE3 38.3 29 107 1124 50

25 NC_017262.1 *Zymomonas mobilis*  46.0 38 182 1497 82

26 NC_020299.1 *Candid.* *Kinetoplastibacterium oncopeltii* 31.2 20 162 625 8

27 NC_020411.1 *Hydrogenobaculum* sp. HO 34.8 27 93 954 34

28 NC_020417.1 Beta proteobacterium CB 46.1 39 90 1697 56

29 NC_021291.1 *Spiribacter salinus* M19-40 62.7 63 80 1151 53

30 NC_022440.1 *Chlamydia pecorum* W73 41.1 35 118 745 22

31 NC_023003.1 *Candidatus babela massiliensis*  27.4 20 159 888 33

**Supplemental Table S2.** Sequences of synthetic DNAs used in this study.

| Name | Sequence (5′ to 3′) | Annotation |
| --- | --- | --- |
| WT | TACGAC**GAATTC**CAAAAATAATG**TTGATCCTTTTAAATAAGTCTGATAAAATGTGAACTAA**AUCUCGCCCAUCCGCUGCCCCAUGGGACUUCCAACCGCAAGGCAGCUUUUUUCACCGUAGGCGCAAGCCACUUGGAGGUCCCUUGAGUUGCACAAGCAUCAUCGCGCGCU**GGATCC**AAAGGA | The *odc1* reporter with *lysC* promoter for making the wild-type reporter construct plasmid, from EcoRI to BamHI restriction sites. Restriction sites are bolded, lysC promoter is in blue, and *odc1* temple template from *S. echinoides* is highlighted in gray. The two alternative start codons are highlighted in red. Nucleotides before and after the restriction enzyme sites are fillers for PCR. |
| M1 | TACGAC**GAATTC**CAAAAATAATG**TTGATCCTTTTAAATAAGTCTGATAAAATGTGAACTAA**AUCUCGCCCAUCCGCUGCCCCAUGGGACUUCCAACCGCAAGGCAGCUUUUUUCACCGUAGGCGCAAGCCACUUGGAGGUCCCUUGAGUUCCACAAGCAUCAUCGCGCGCU**GGATCC**AAAGGA | The template for making the M1 mutant *odc1* reporter. Annotations are described above. |
| M2 | TACGAC**GAATTC**CAAAAATAATG**TTGATCCTTTTAAATAAGTCTGATAAAATGTGAACTAA**AUCUCGCCCAUCCGCUGCCCCAUGGGACUUCCAACCGCAAGGCAGCUUUUUUCACCGUAGGCGCAAGCCACUUGGAGGUCCCUUCAGUUGCACAAGCAUCAUCGCGCGCU**GGATCC**AAAGGA | The template for making the M2 mutant *odc1* reporter. Annotations are described above. |
| M3 | TACGAC**GAATTC**CAAAAATAATG**TTGATCCTTTTAAATAAGTCTGATAAAATGTGAACTAA**AUCUCGCCCAUCCGCUGCCCCAUGGGACUUCCAACCGCAAGGCAGCUUUUUUCACCGUAGGCGCAAGCCACUUGGAGGUCCCUUCAGUUCCACAAGCAUCAUCGCGCGCU**GGATCC**AAAGGA | The template for making the M3 mutant *odc1* reporter. Annotations are described above. |
| M4 | TACGAC**GAATTC**CAAAAATAATG**TTGATCCTTTTAAATAAGTCTGATAAAATGTGAACTAA**AUCUCGCCCAUCCGCUGCCCCAUGGGACUUCCAACAGCAAGGCAGCUUUUUUCACCGUAGGCGCAAGCCACUUGGAGGUCCCUUGAGUUGCACAAGCAUCAUCGCGCGCU**GGATCC**AAAGGA | The template for making the M4 mutant *odc1* reporter. Annotations are described above. |
| M5 | TACGAC**GAATTC**CAAAAATAATG**TTGATCCTTTTAAATAAGTCTGATAAAATGTGAACTAA**AUCUCGCCCAUCCGCUGCCCCAUGGGACUUCCAACCGAAAGGCAGCUUUUUUCACCGUAGGCGCAAGCCACUUGGAGGUCCCUUCAGUUGCACAAGCAUCAUCGCGCGCU**GGATCC**AAAGGA | The template for making the M5 mutant *odc1* reporter. Annotations are described above. |
| M6 | TACGAC**GAATTC**CAAAAATAATG**TTGATCCTTTTAAATAAGTCTGATAAAATGTGAACTAA**AUCUCGCCCAUCCGCUGCCCCAUGGGACUUCCAACCGCCAGGCAGCUUUUUUCACCGUAGGCGCAAGCCACUUGGAGGUCCCUUCAGUUGCACAAGCAUCAUCGCGCGCU**GGATCC**AAAGGA | The template for making the M6 mutant *odc1* reporter. Annotations are described above. |

PCR Forward Primer

TAC GAC GAA TTC CAA AAA TAA TGT TGA TCC (56.1)

PCR Reverse Primer

TCC TTT GGA TCC AGC GC (55.3)

PCR Reverse Primer (For making +1 frame reporter constructs)

TCC TTT GGA TCC CAG CGC


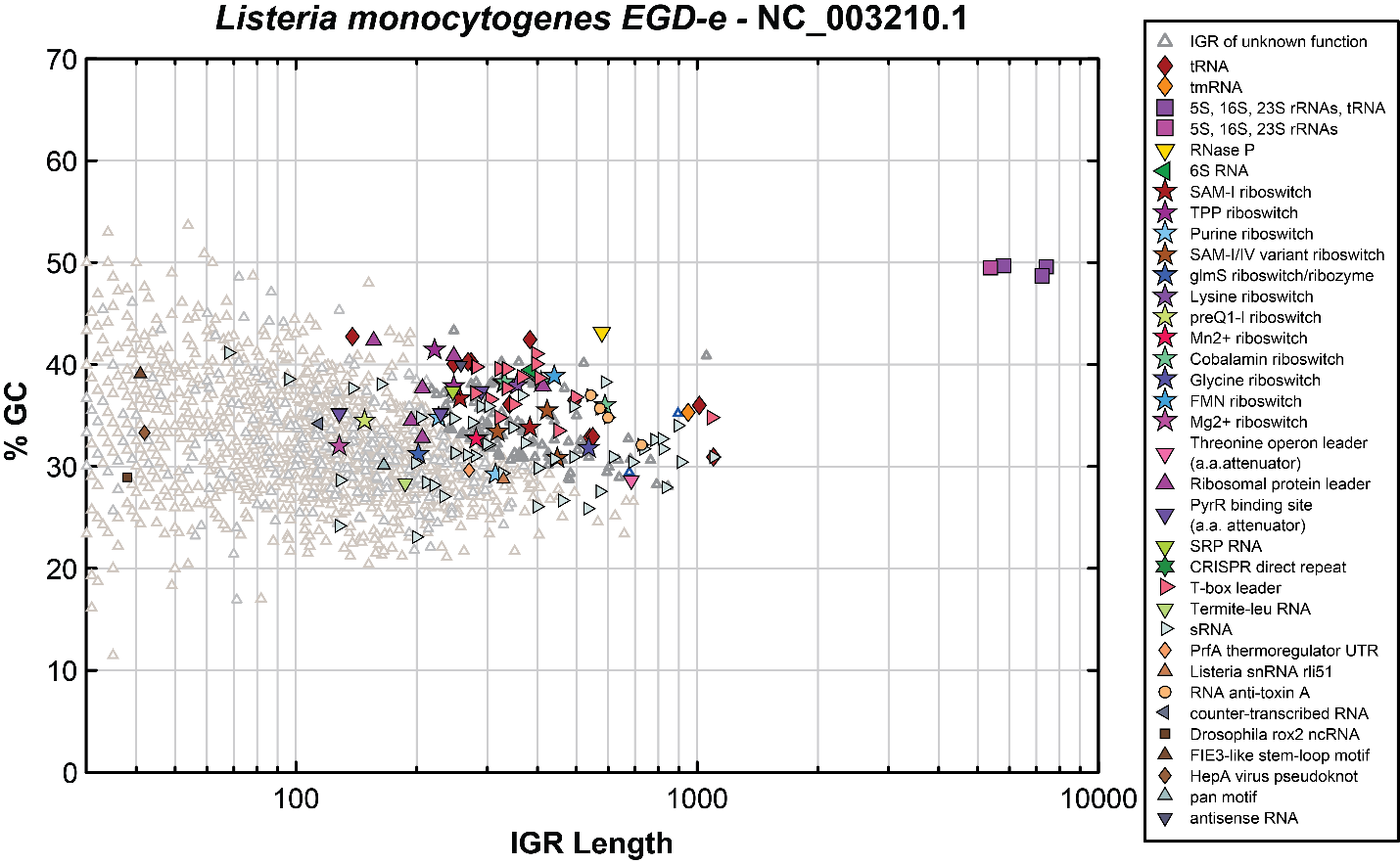


**Fig S1.** Plots of the IGRs from the *L. monocytogenes* genome sorted by IGR length and GC content.


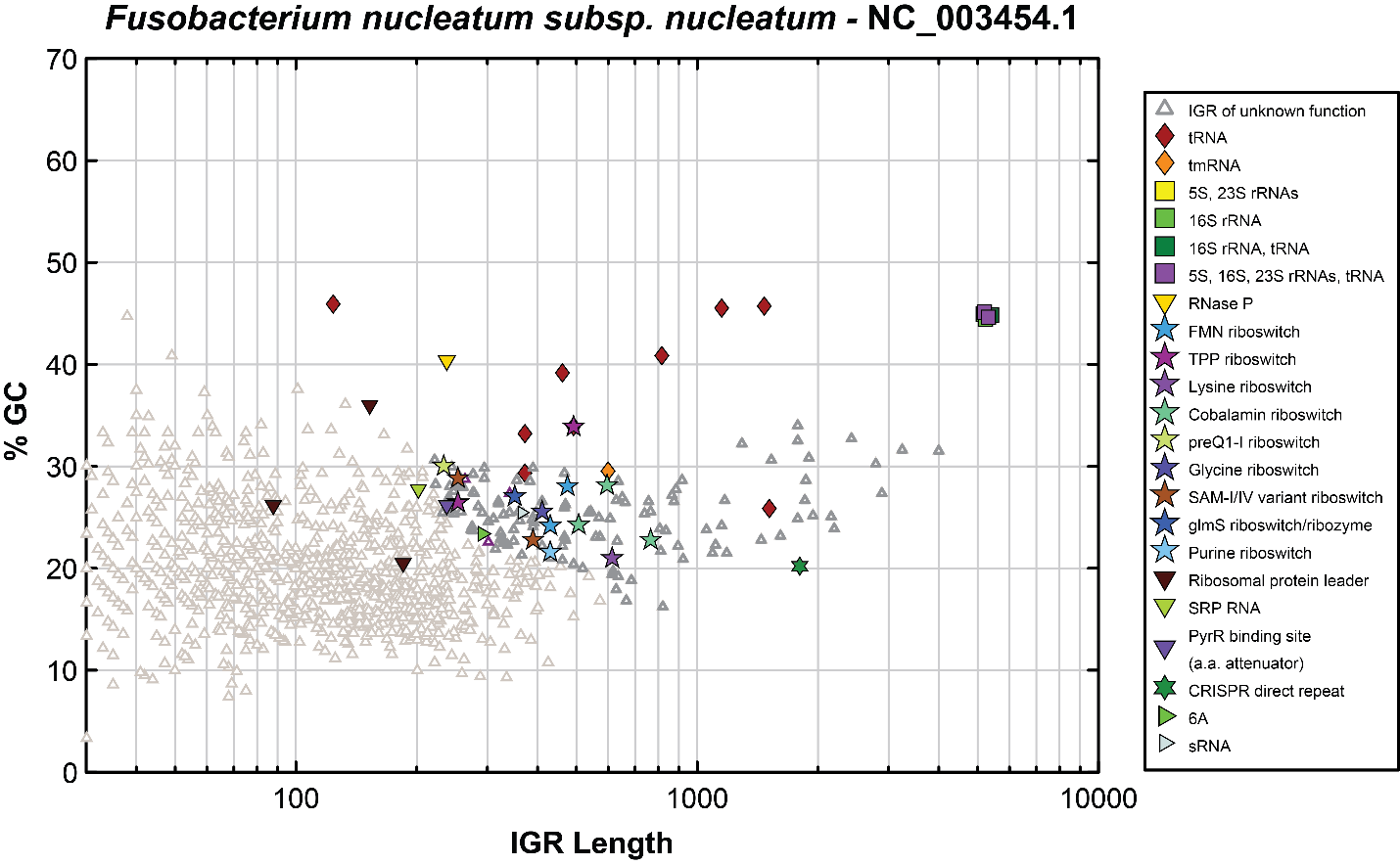


**Fig S2.** Plots of the IGRs from the *F. nucleatum* genome sorted by IGR length and GC content.

**
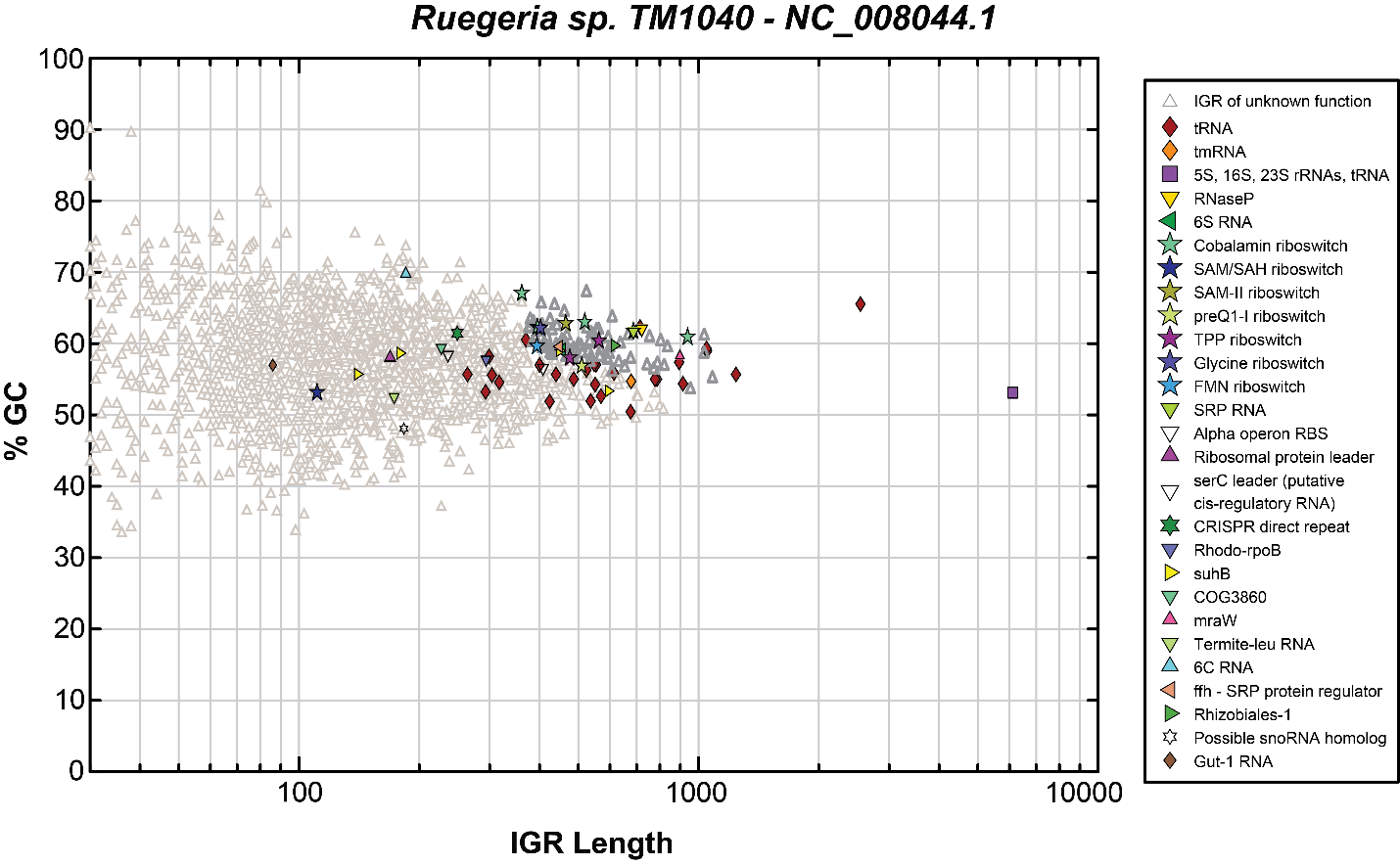
**

**Fig S3.** Plots of the IGRs from the *Ruegeria* genome sorted by IGR length and GC content.


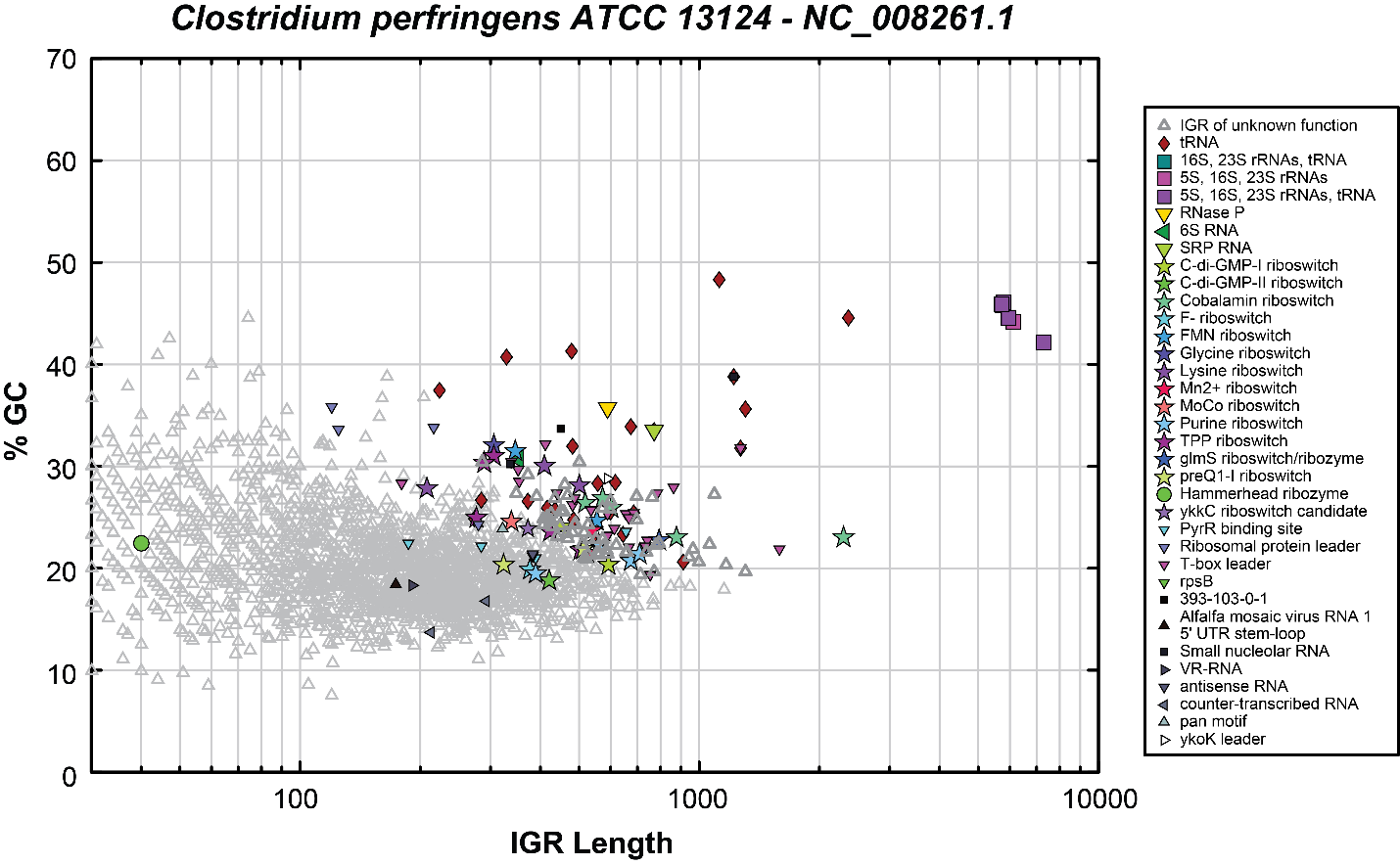


**Fig S4.** Plots of the IGRs from the *C. perfringens* genome sorted by IGR length and GC content


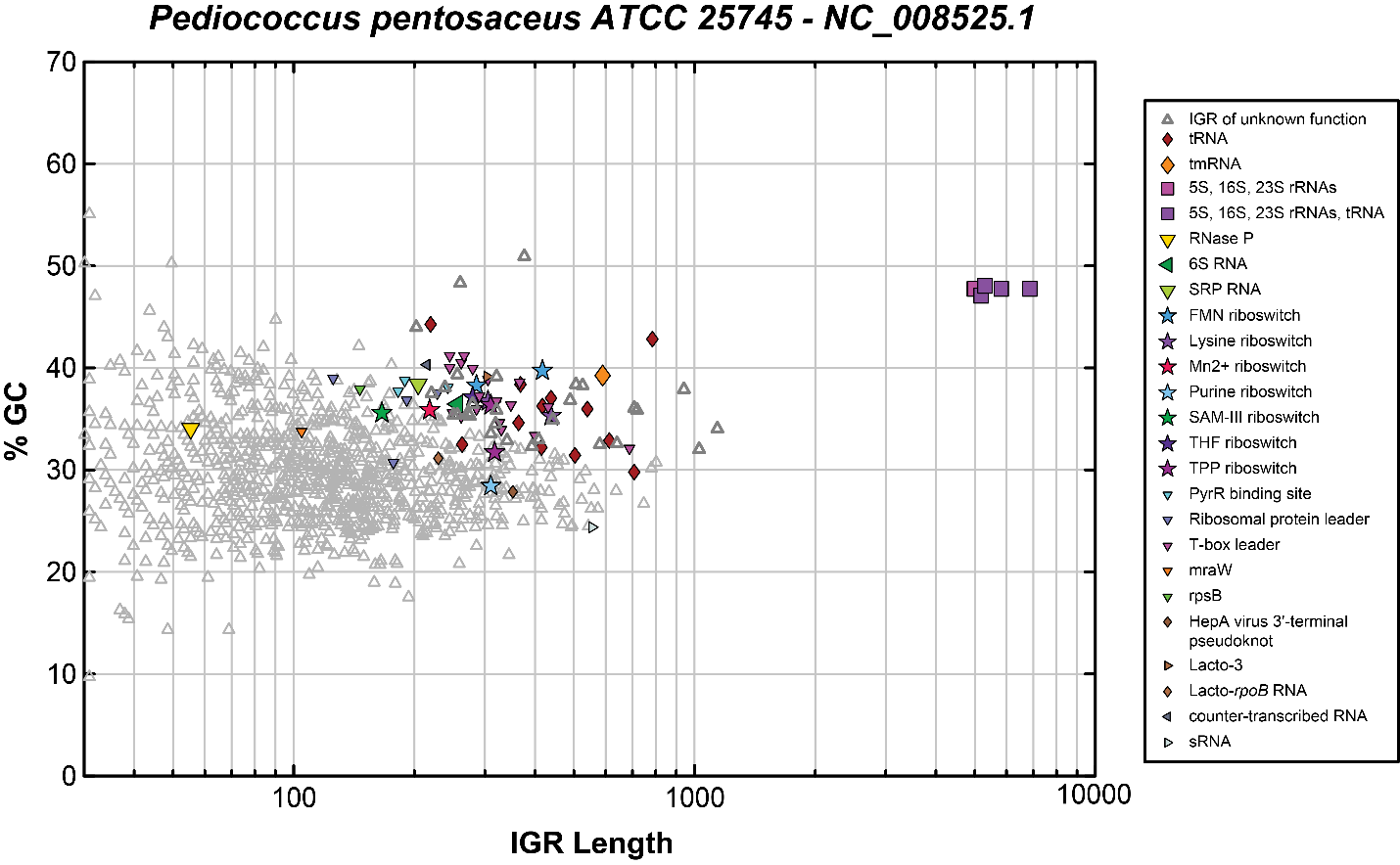


**Fig S5.** Plots of the IGRs from the *P. pentosaceus* genome sorted by IGR length and GC content.


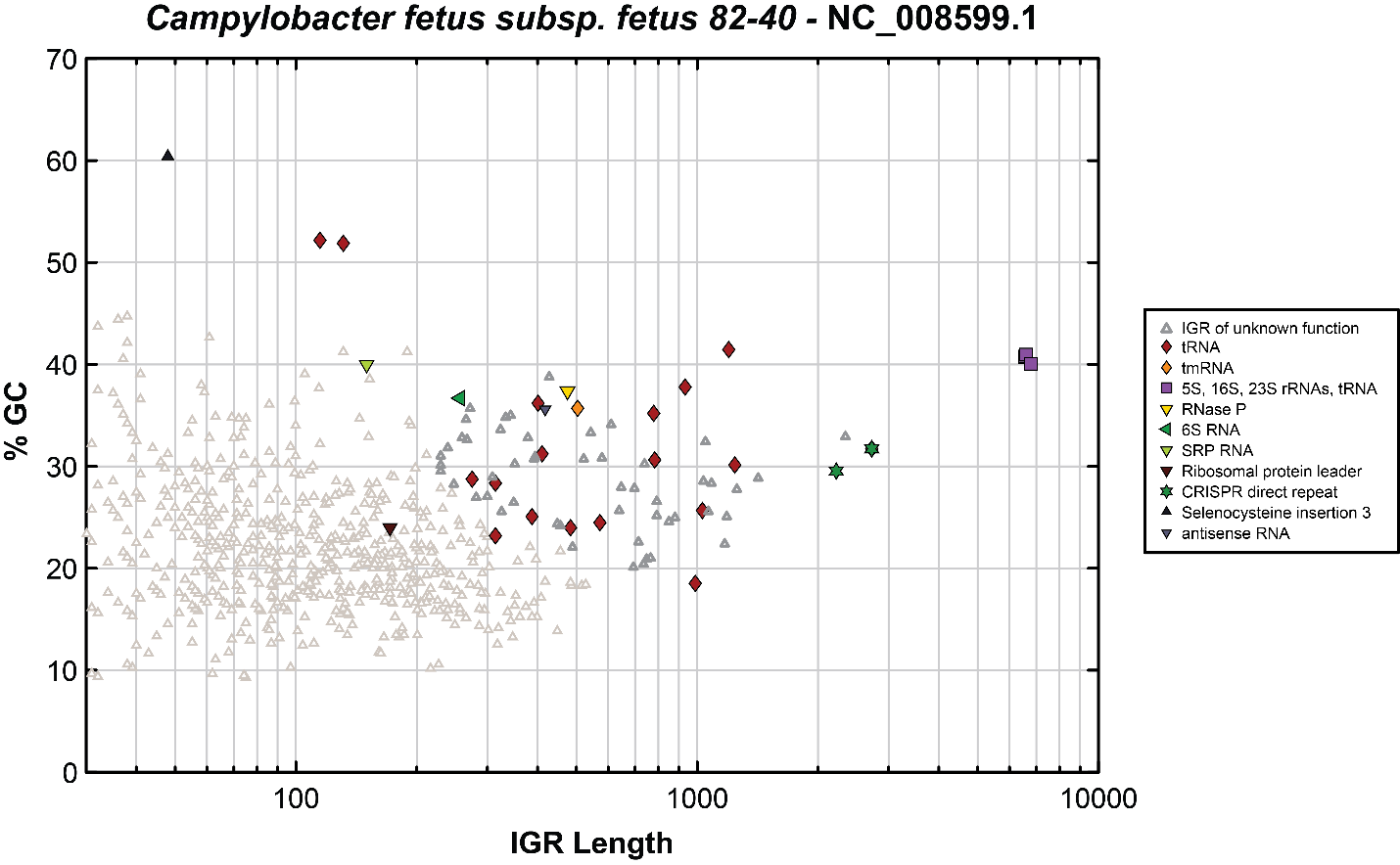


**Fig S6.** Plots of the IGRs from the *C. fetus* genome sorted by IGR length and GC content.


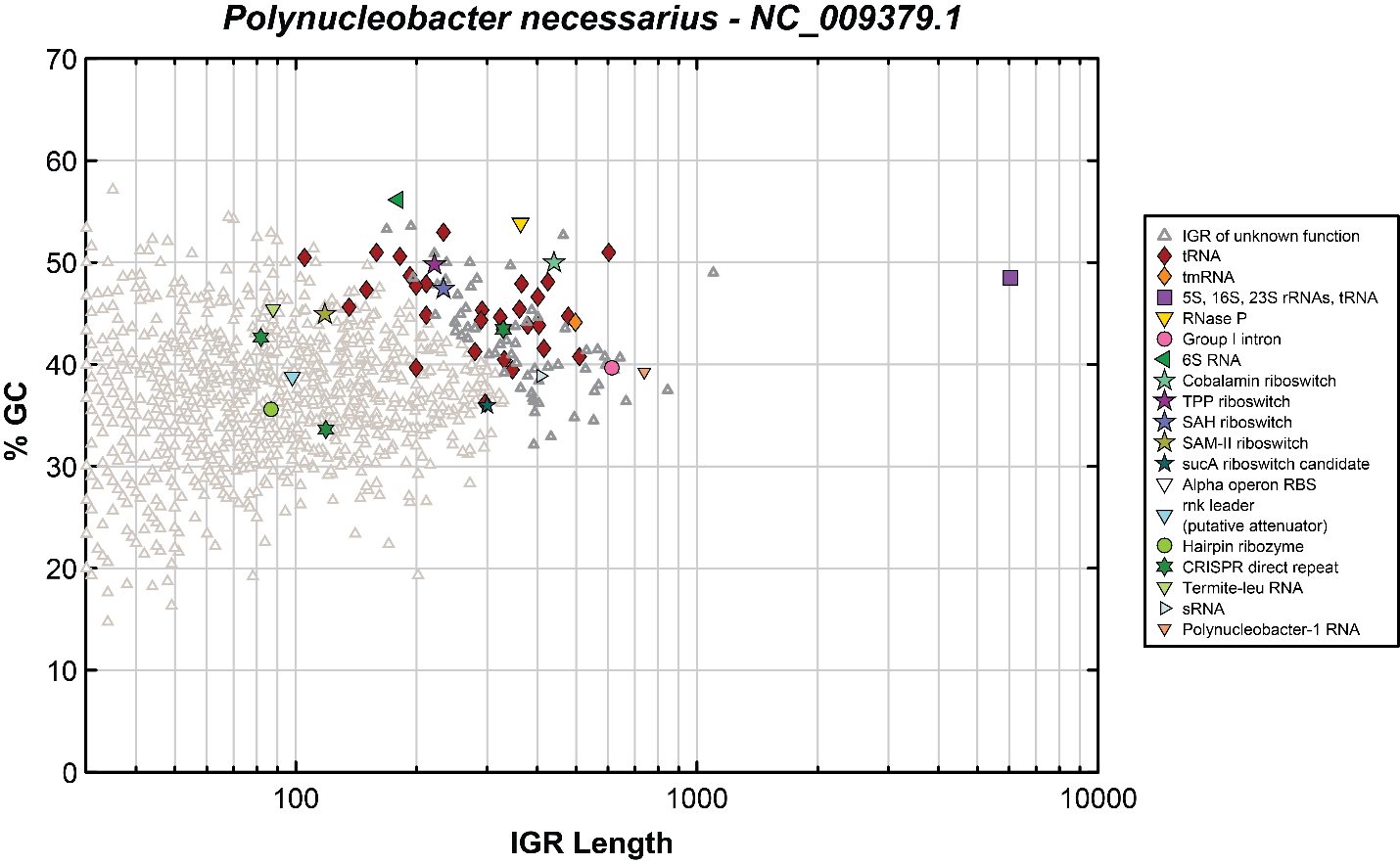


**Fig S7.** Plots of the IGRs from the P*. necessarius* genome sorted by IGR length and GC content.


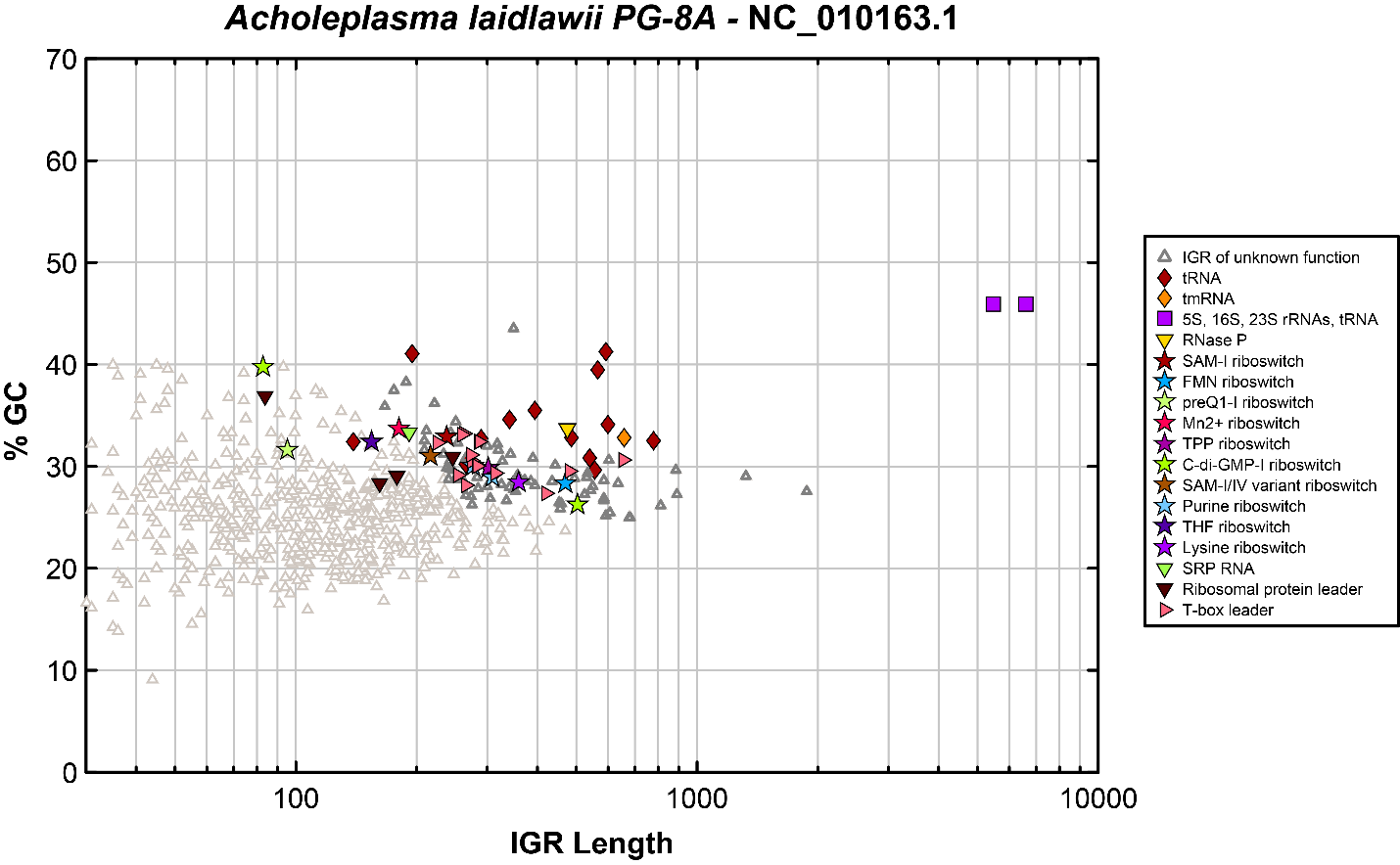


**Fig S8.** Plots of the IGRs from the *A. laidlawii* genome sorted by IGR length and GC content.


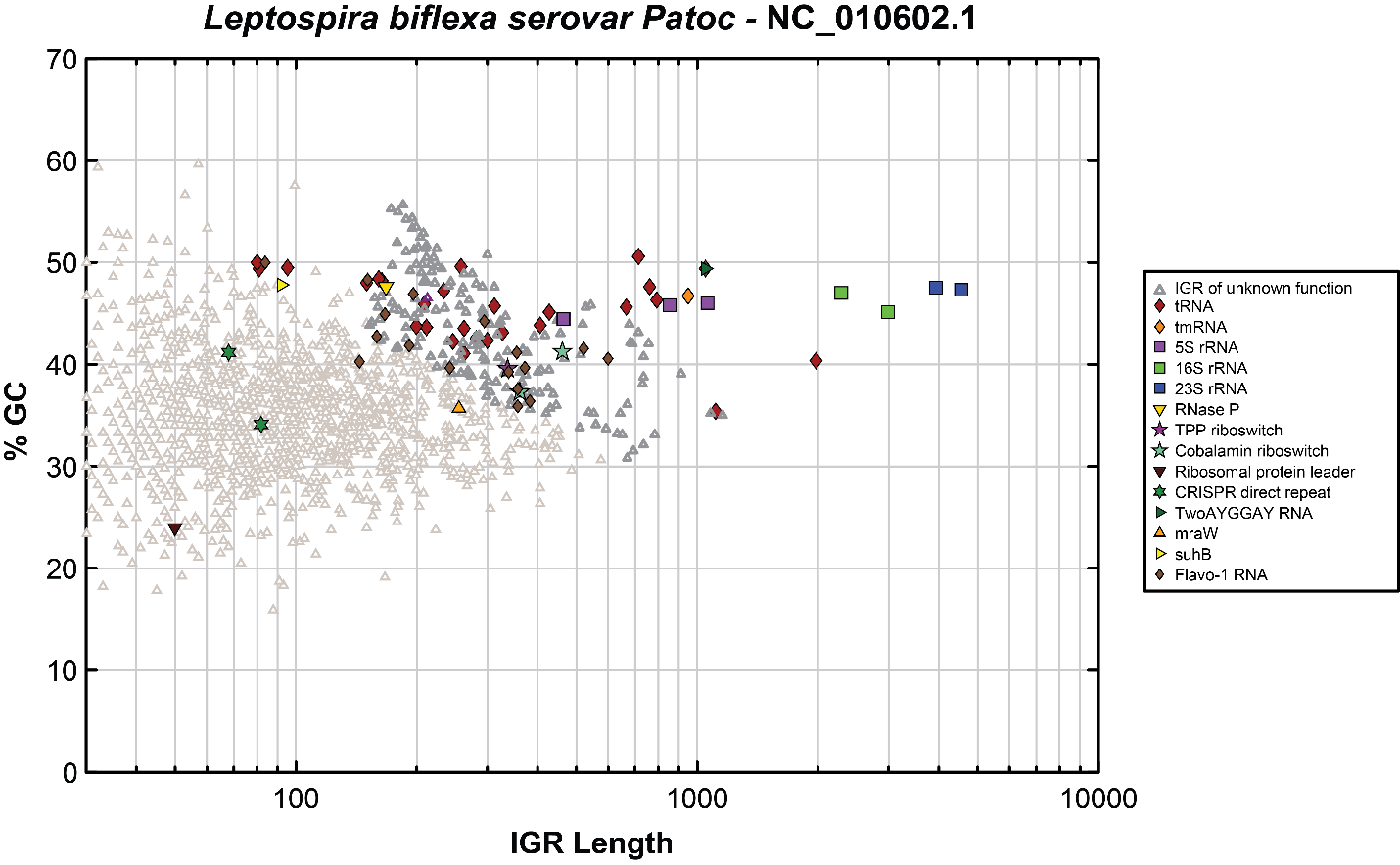


**Fig S9.** Plots of the IGRs from the *L. biflexa* genome sorted by IGR length and GC content.


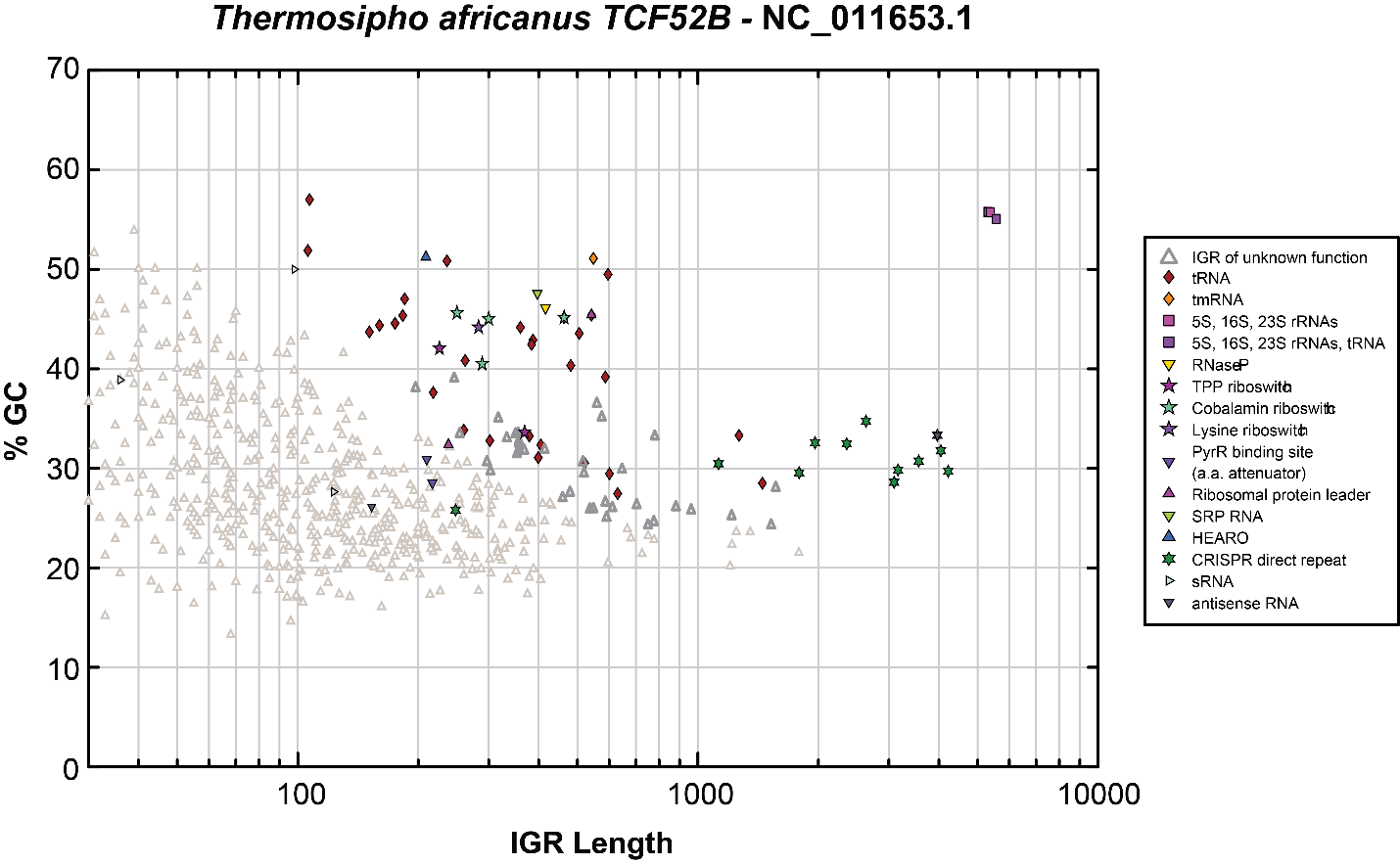


**Fig S10.** Plots of the IGRs from the *T. africanus* genome sorted by IGR length and GC content.


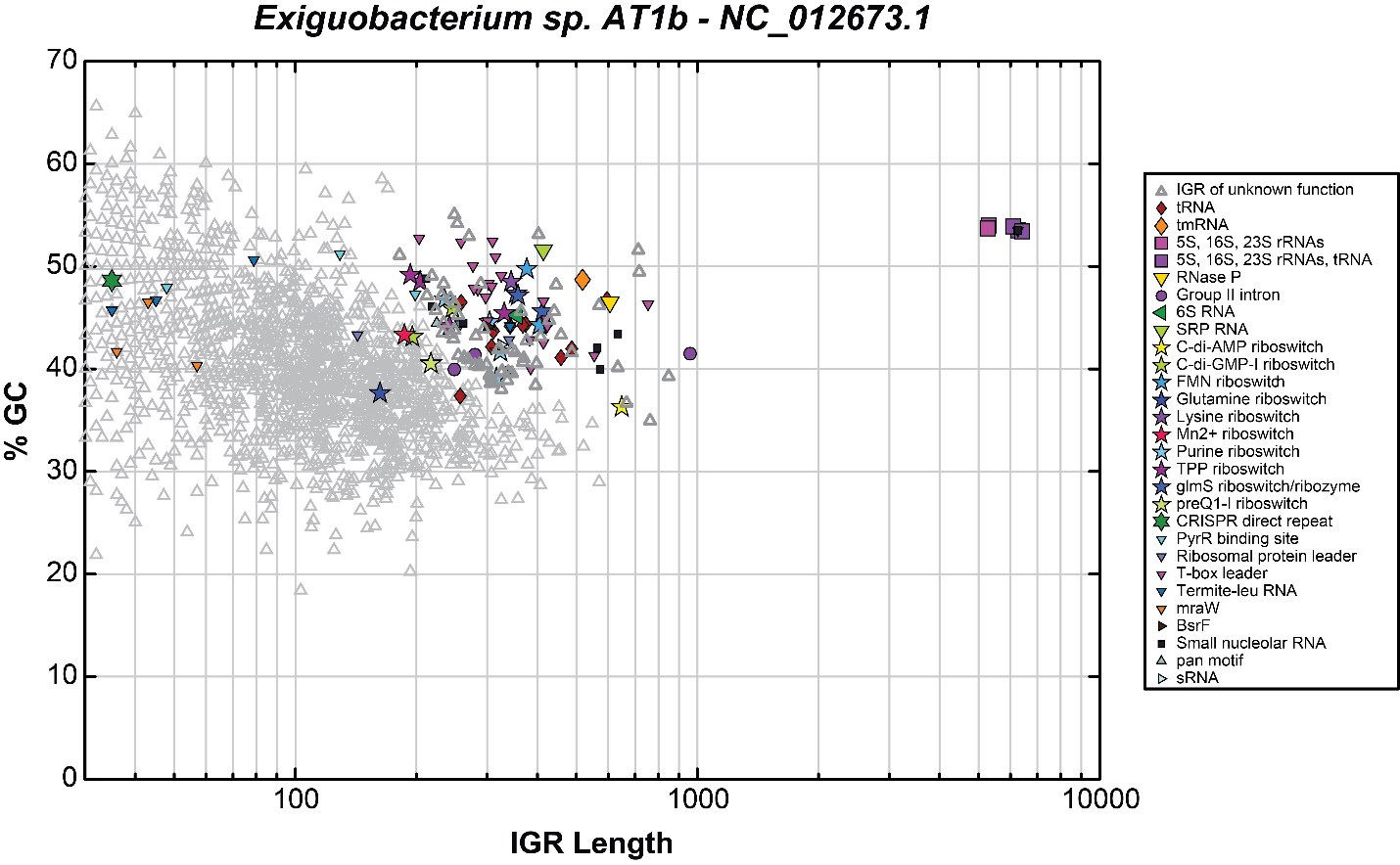


**Fig S11.** Plots of the IGRs from the *Exiguobacterium sp. AT1b* genome sorted by IGR length and GC content.

**
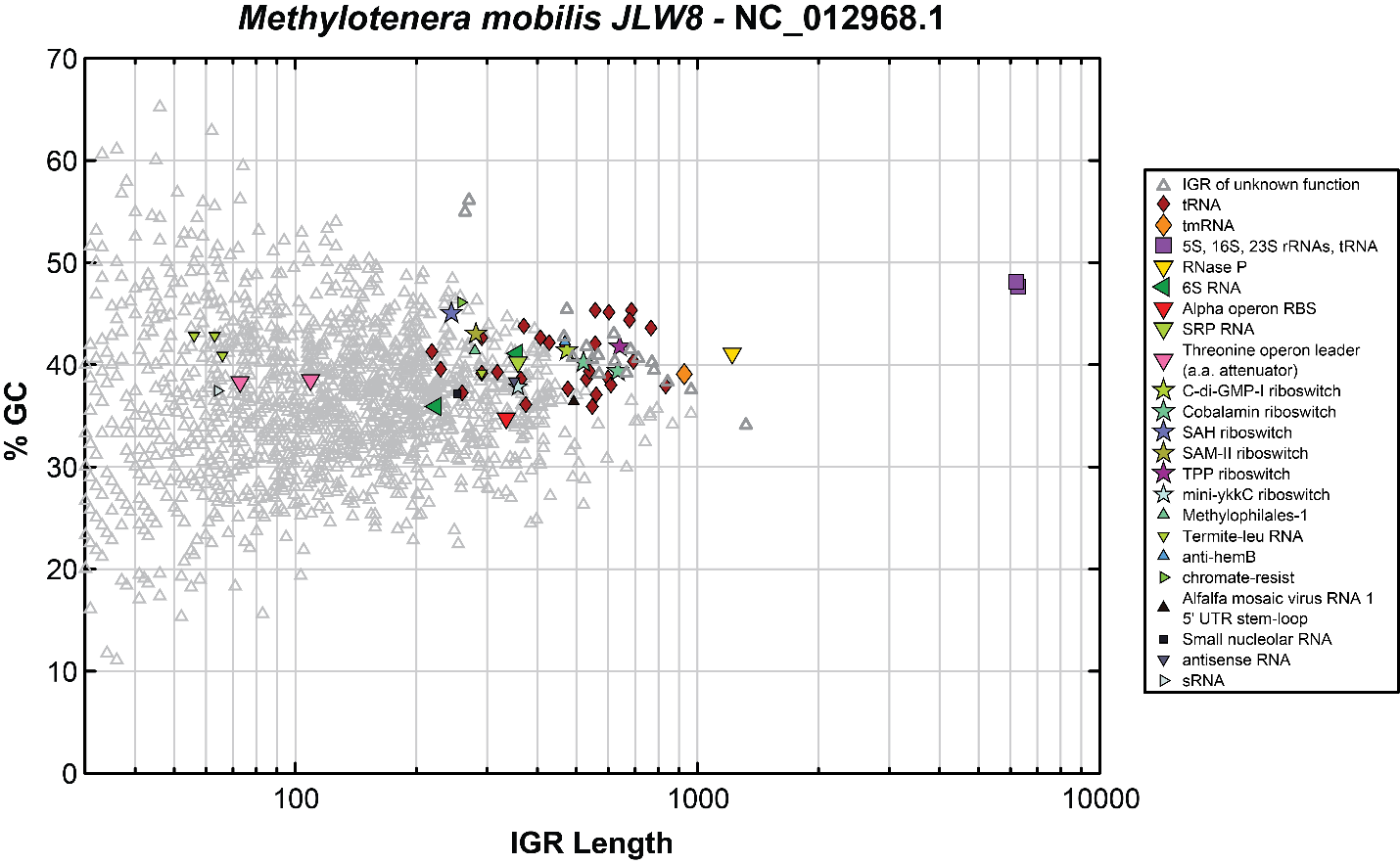
**

**Fig S12.** Plots of the IGRs from the *M. mobilis* genome sorted by IGR length and GC content.

**
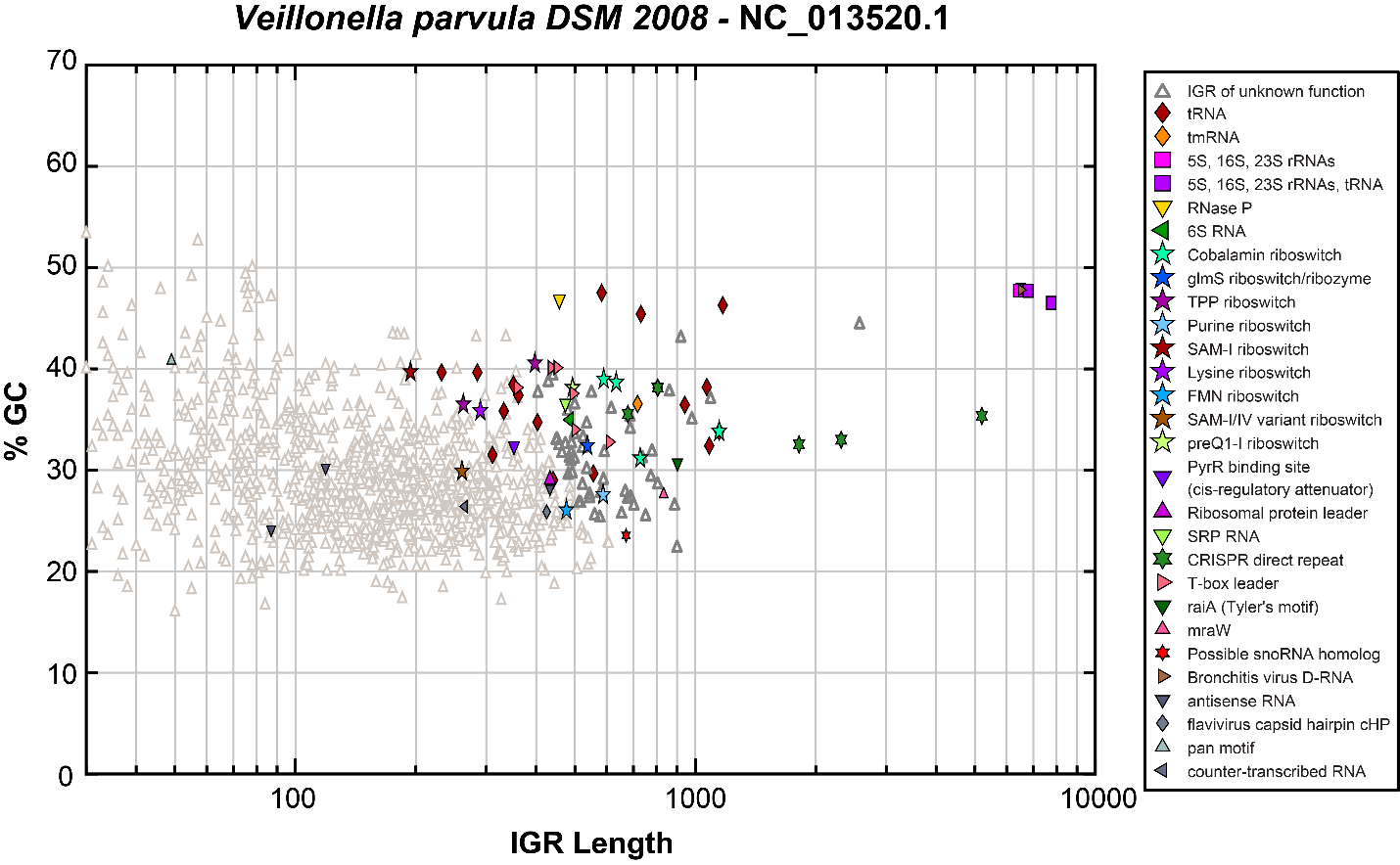
**

**Fig S13.** Plots of the IGRs from the *V. parvula* genome sorted by IGR length and GC content.


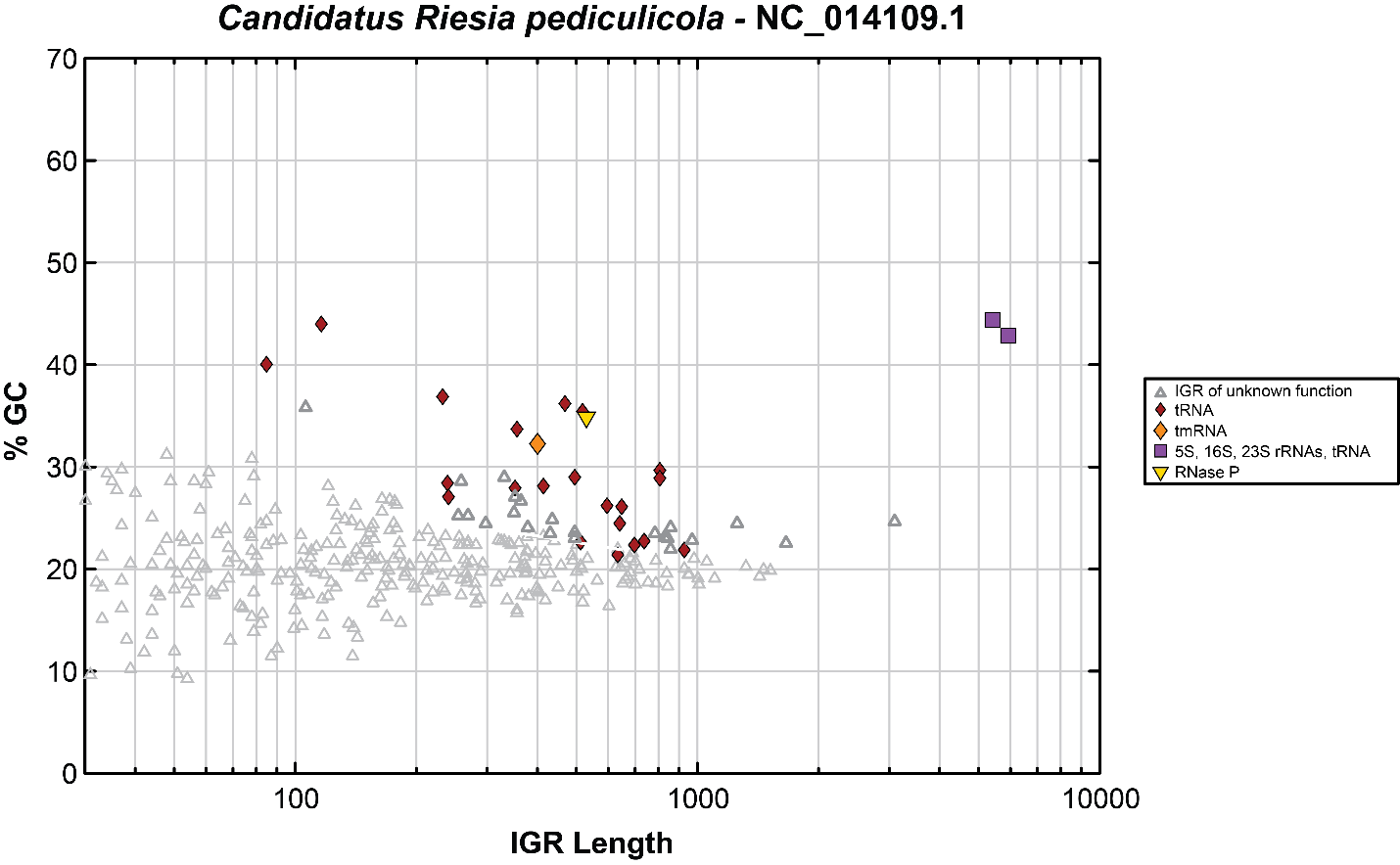


**Fig S14.** Plots of the IGRs from the *C. R. pediculicola* genome sorted by IGR length and GC content.


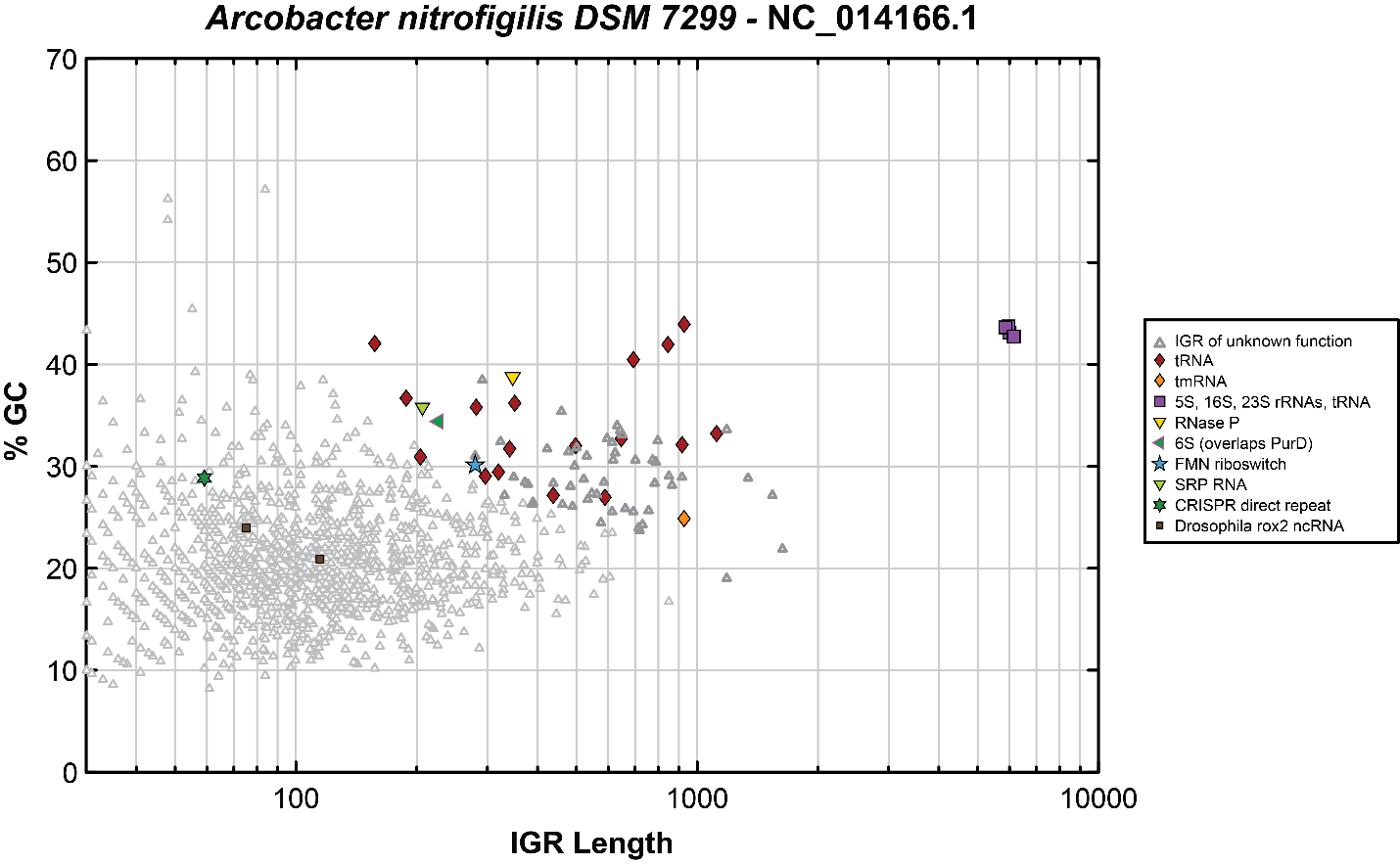


**Fig S15.** Plots of the IGRs from the *A. nitrofigilis DSM 7299* genome sorted by IGR length and GC content.


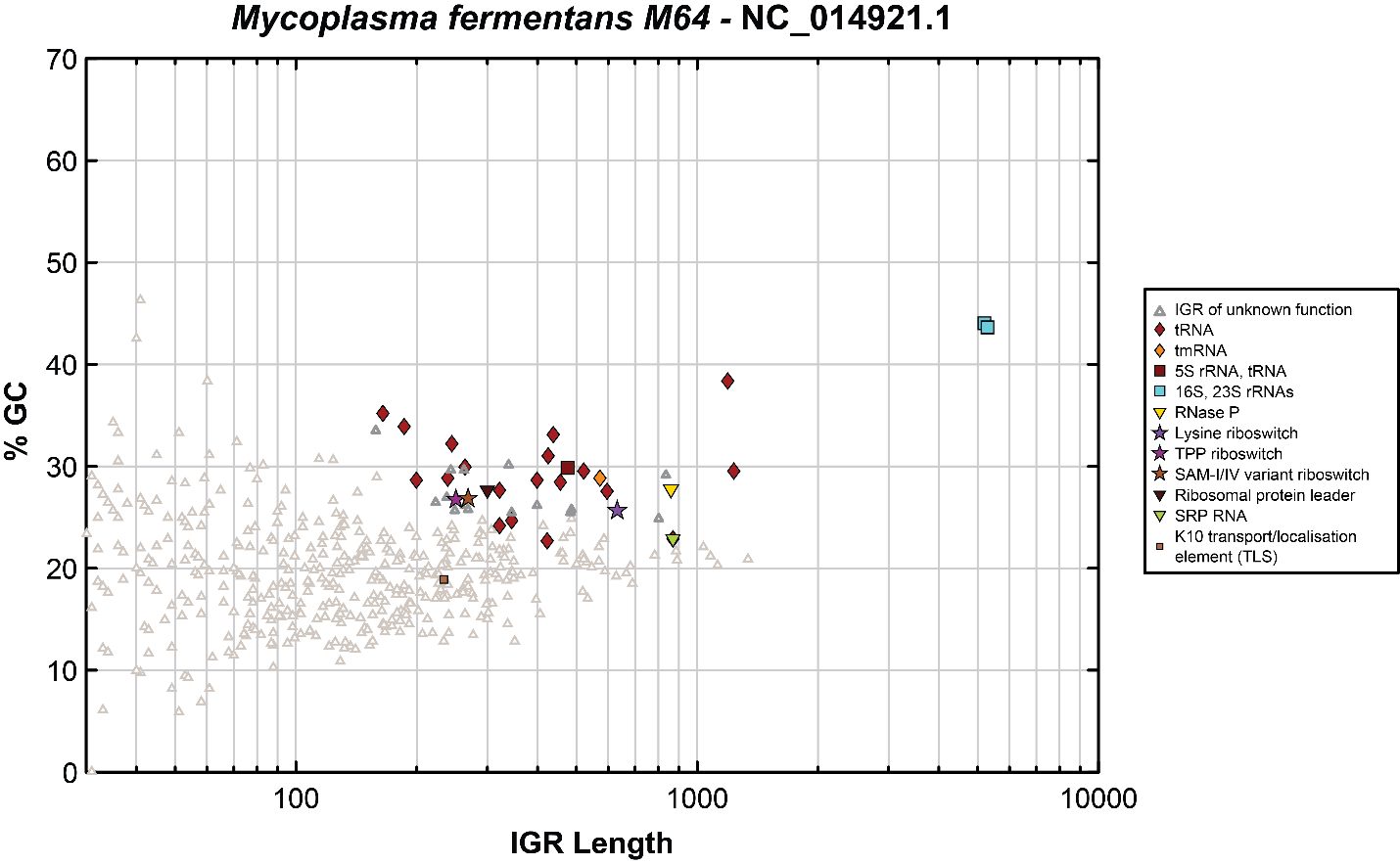


**Fig S16.** Plots of the IGRs from the *M. fermentans* genome sorted by IGR length and GC content.


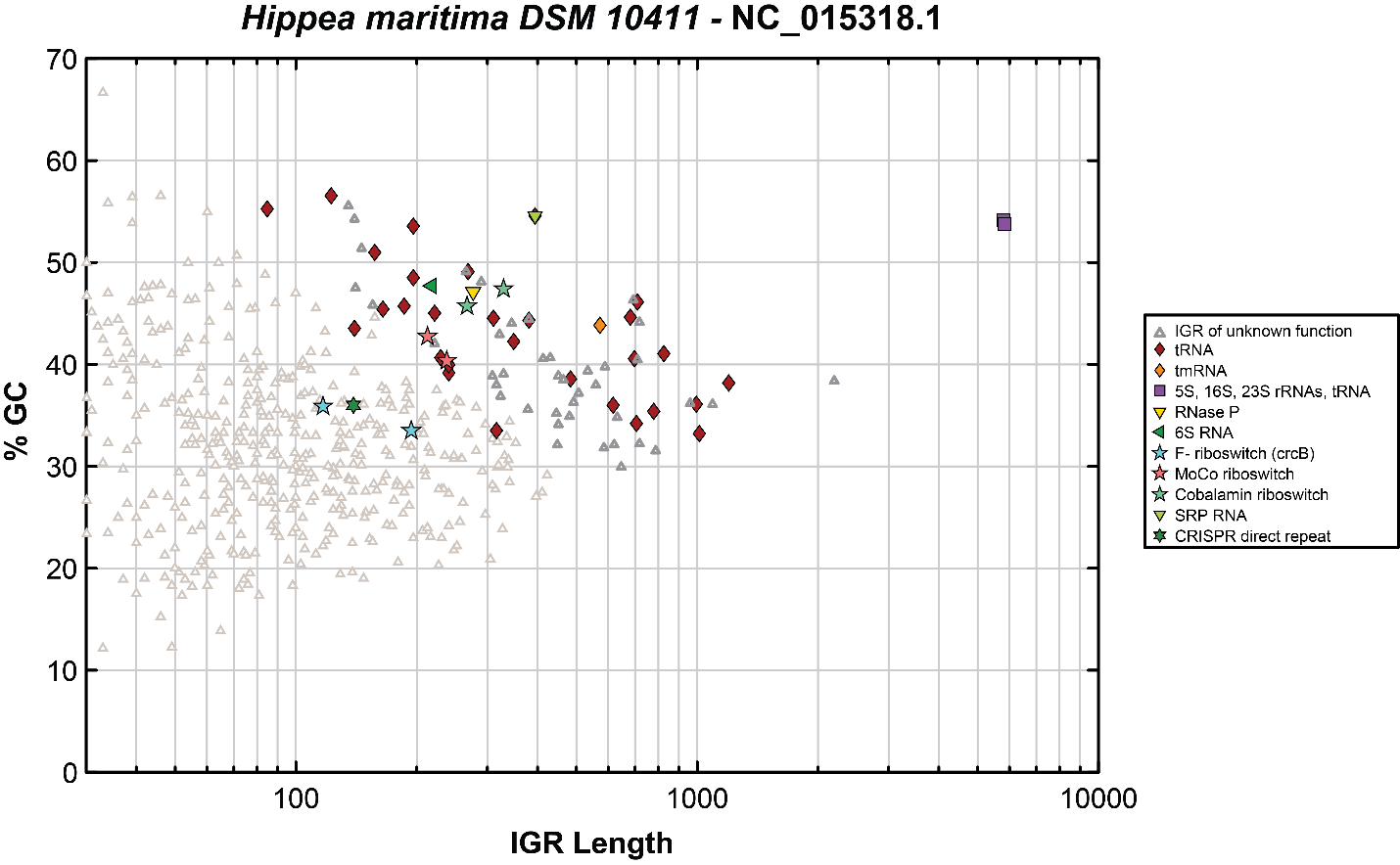


**Fig S17.** Plots of the IGRs from the *H.* *maritima* genome sorted by IGR length and GC content.


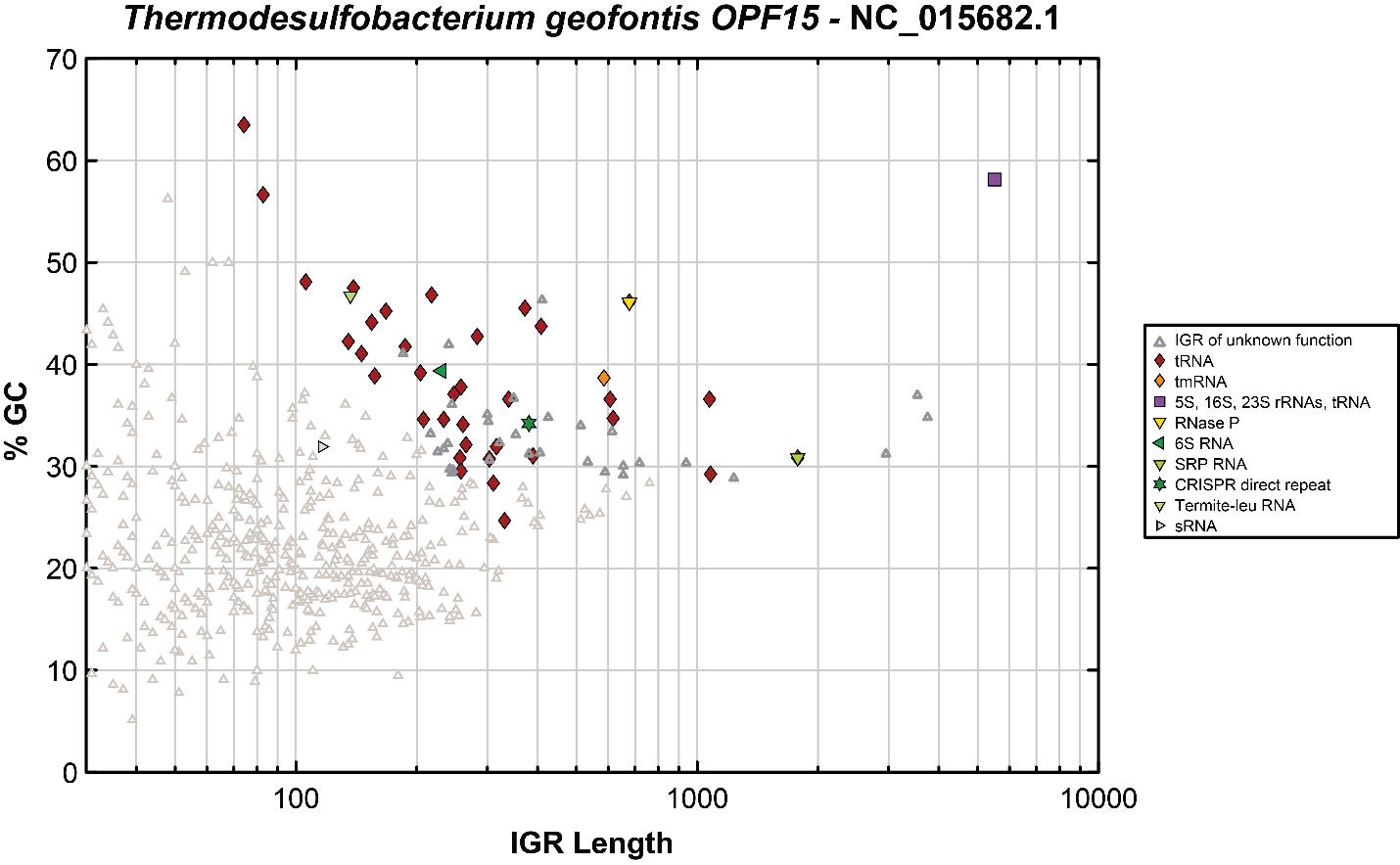


**Fig S18.** Plots of the IGRs from the *T. geofontis* genome sorted by IGR length and GC content.


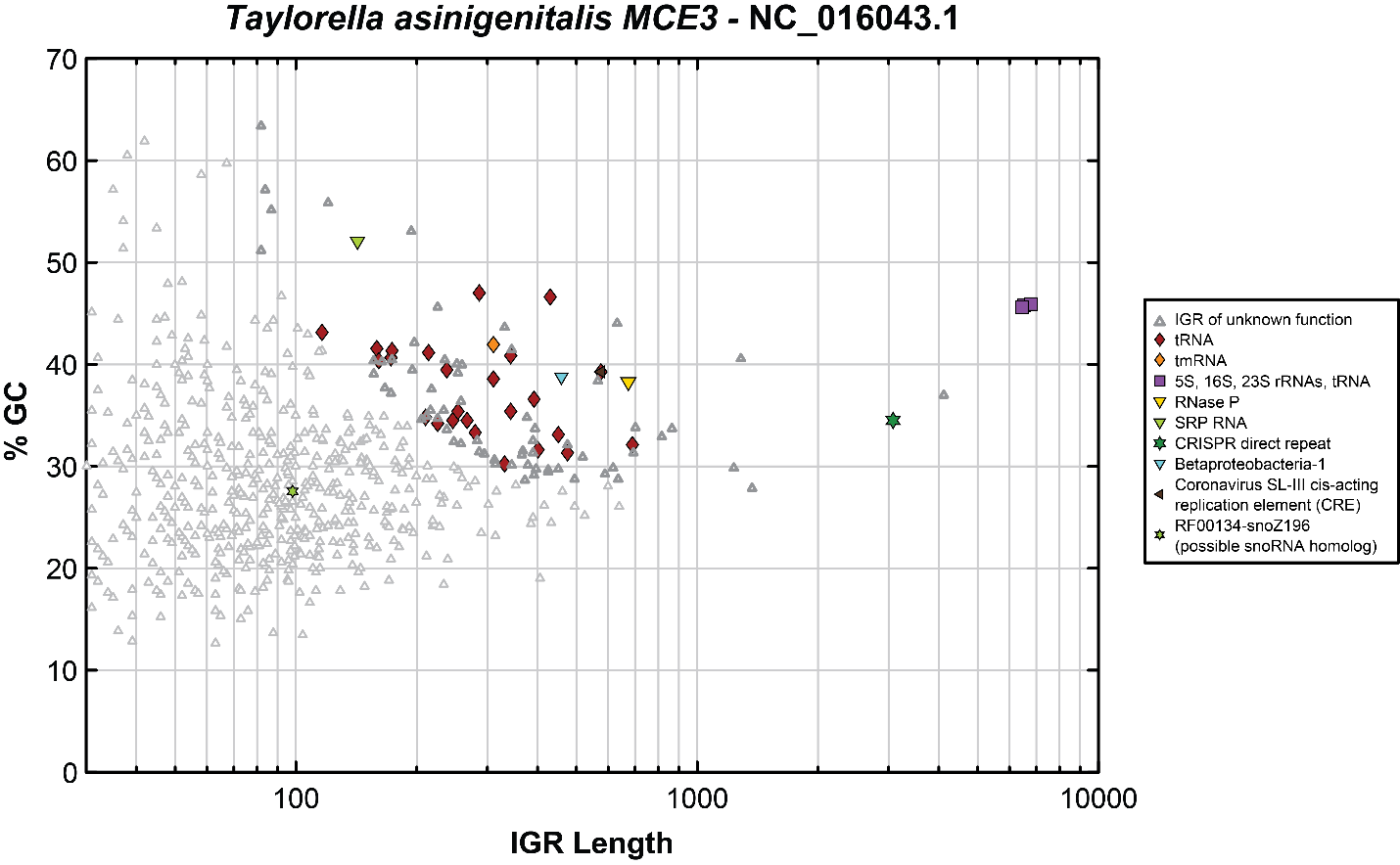


**Fig S19.** Plots of the IGRs from the *T. asinigenitalis MCE3* genome sorted by IGR length and GC content.


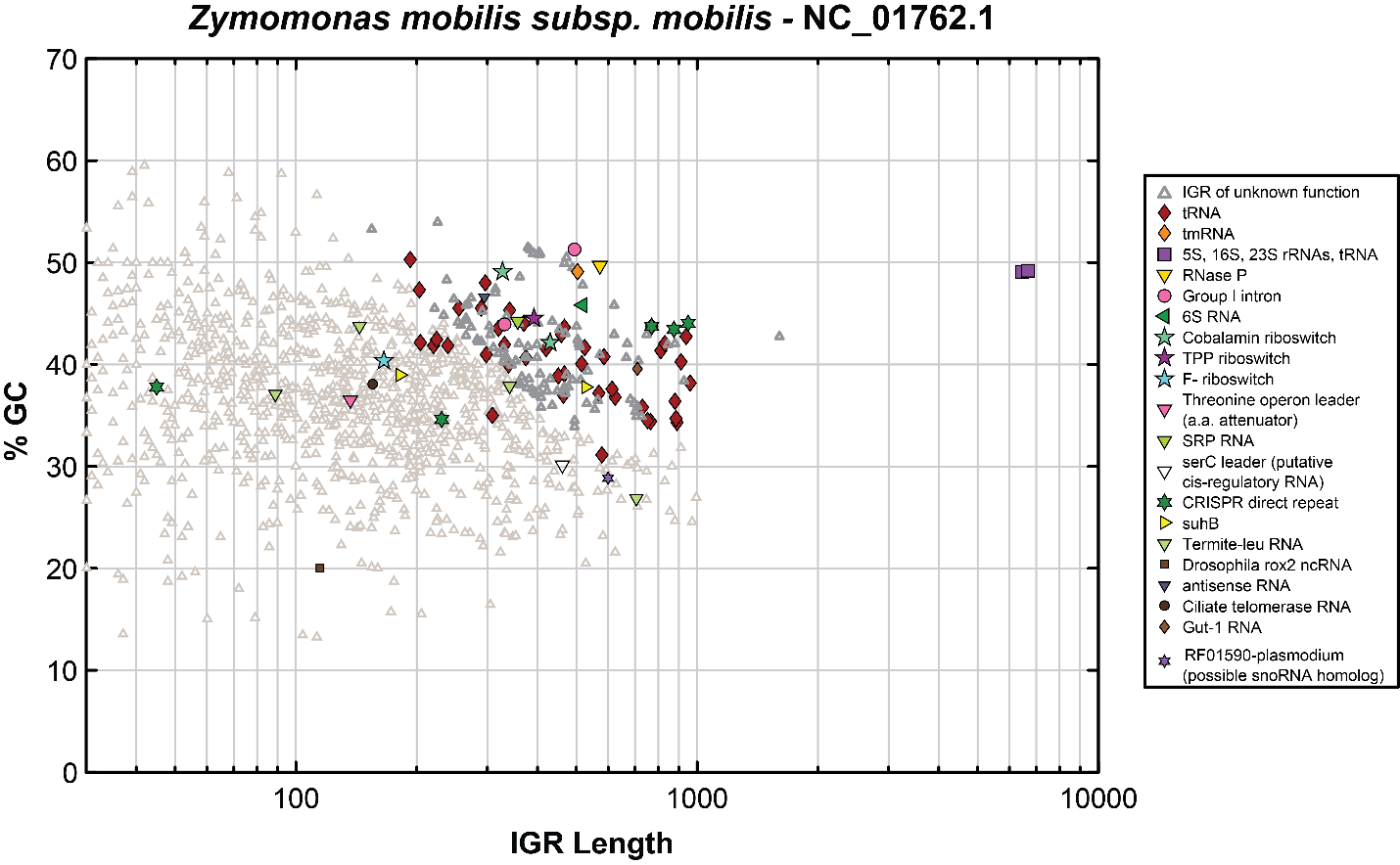


**Fig S20.** Plots of the IGRs from the *Z. mobilis* genome sorted by IGR length and GC content.


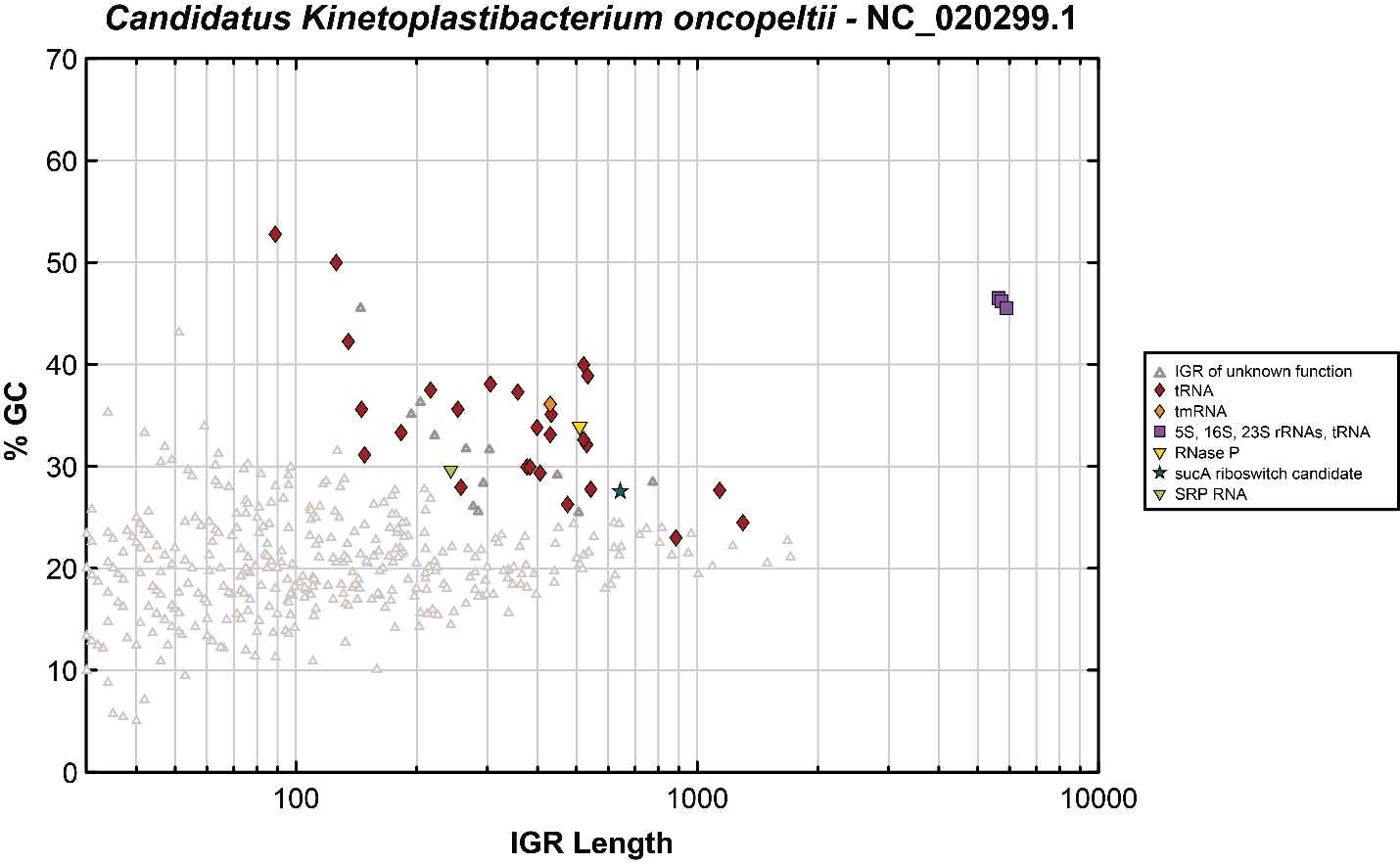


**Fig S21.** Plots of the IGRs from the *C. K. oncopeltii* genome sorted by IGR length and GC content.


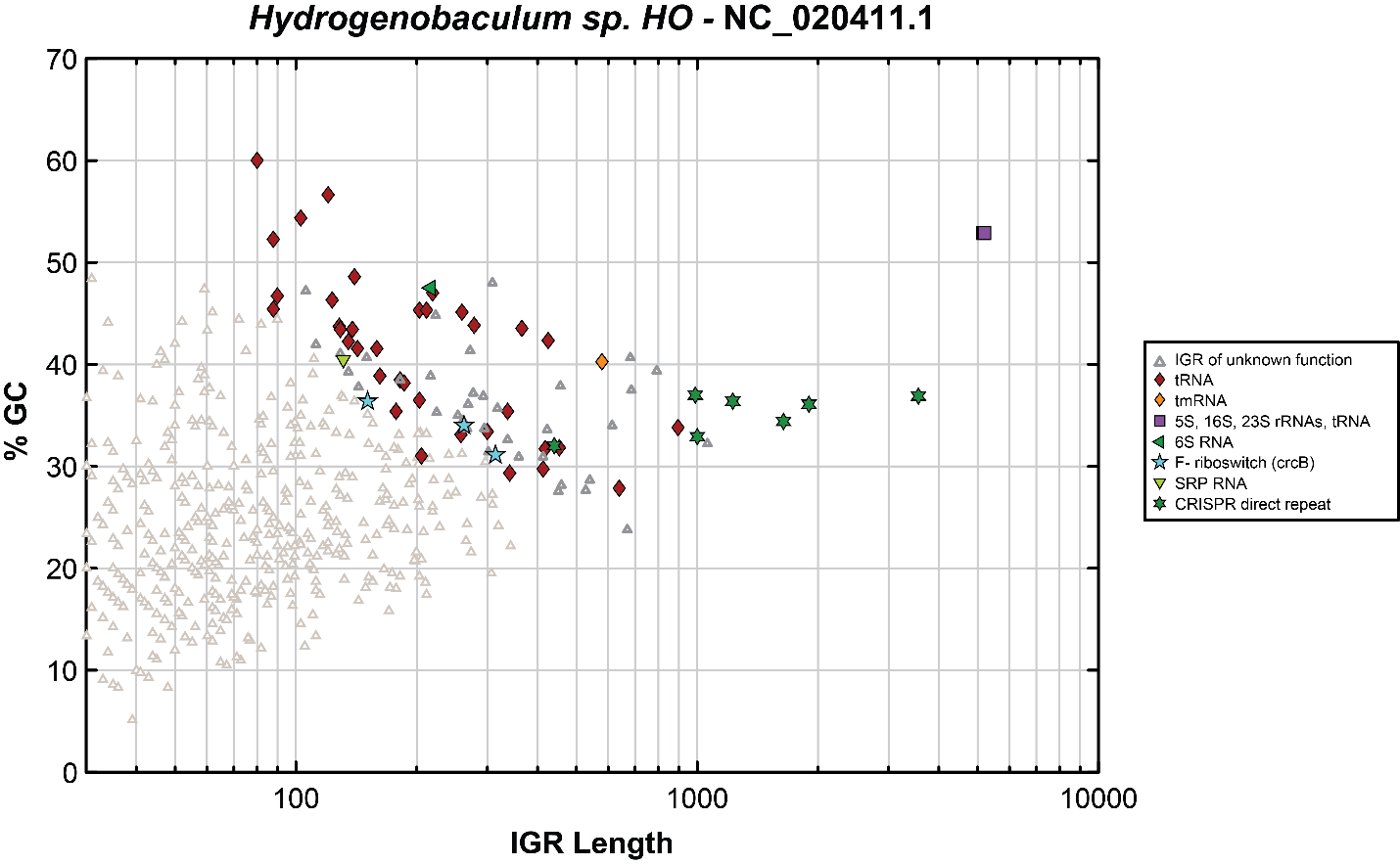


**Fig S22.** Plots of the IGRs from the *H.* sp. HO genome sorted by IGR length and GC content.


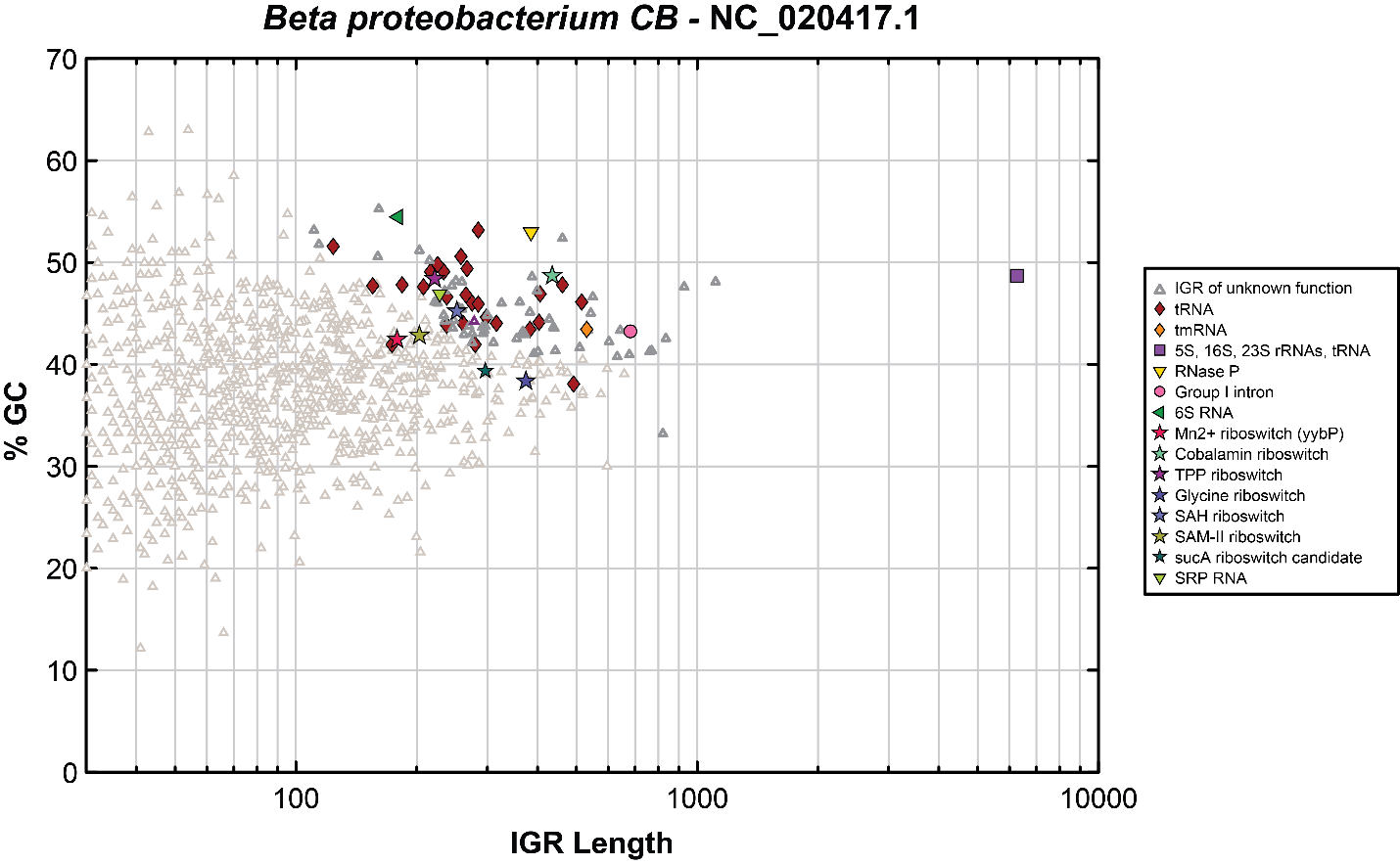


**Fig S23.** Plots of the IGRs from the *B. proteobacterium* genome sorted by IGR length and GC content.
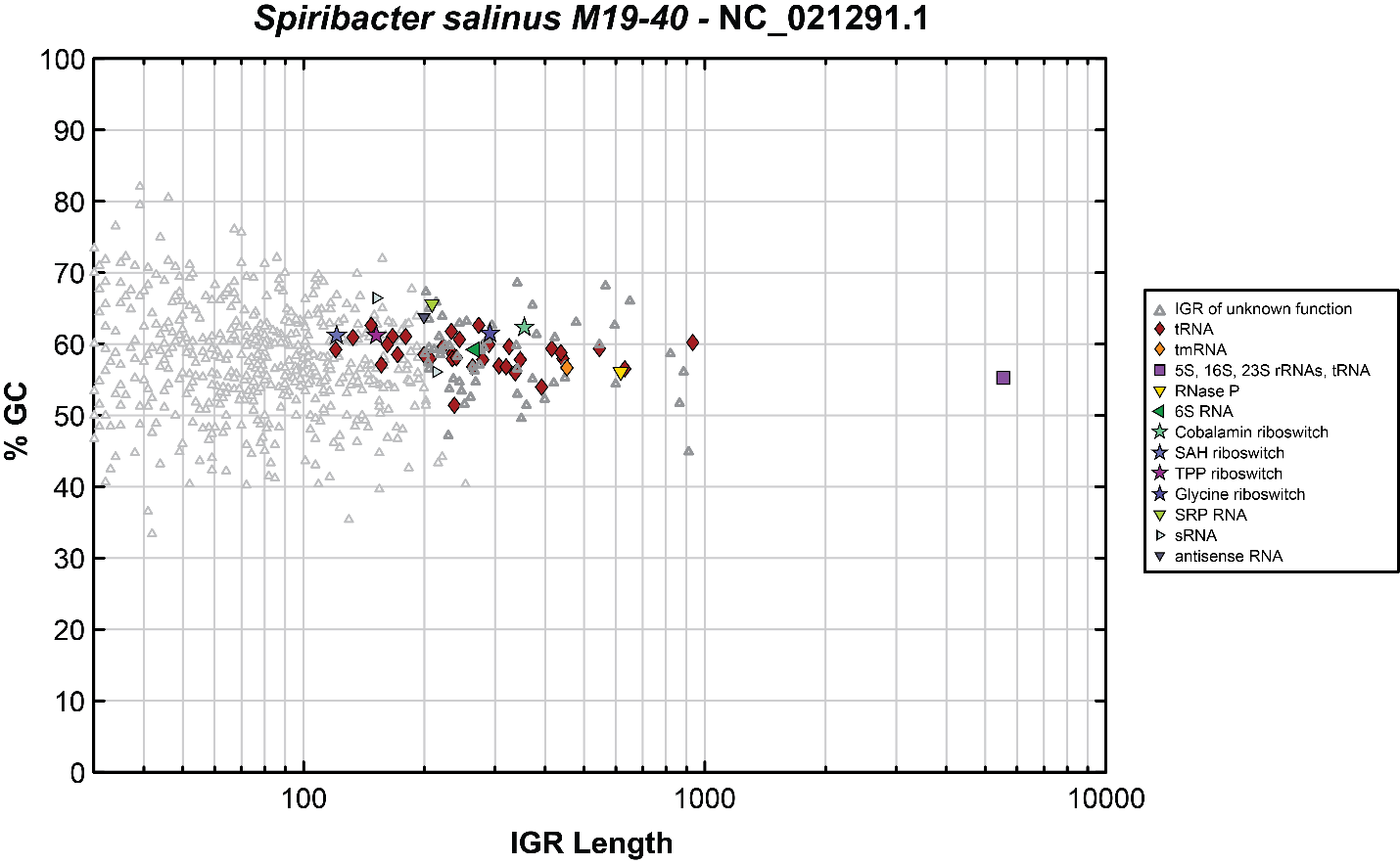


**Fig S24.** Plots of the IGRs from the *S. salinus* genome sorted by IGR length and GC content.


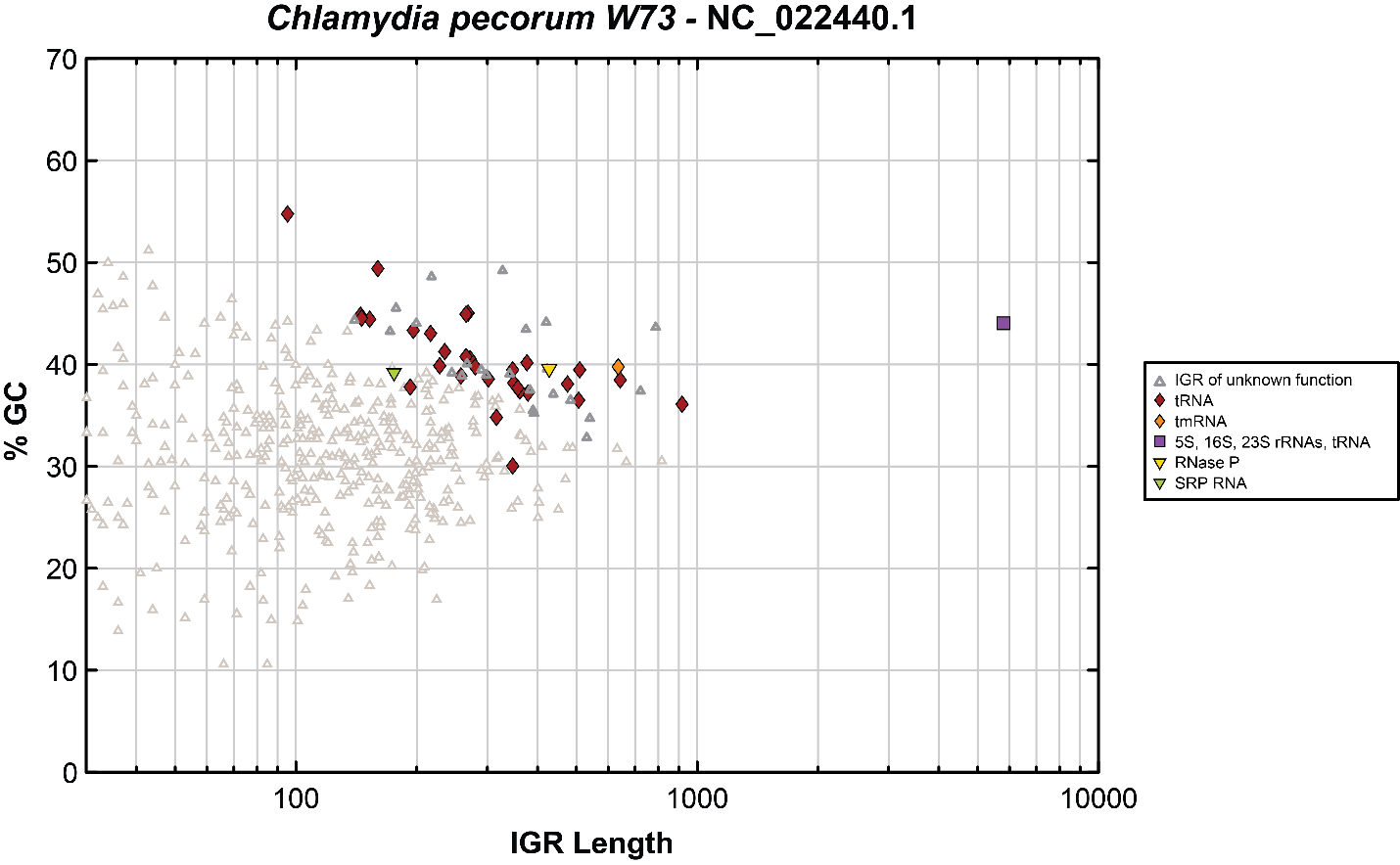


**Fig S25.** Plots of the IGRs from the *C. pecorum* genome sorted by IGR length and GC content.


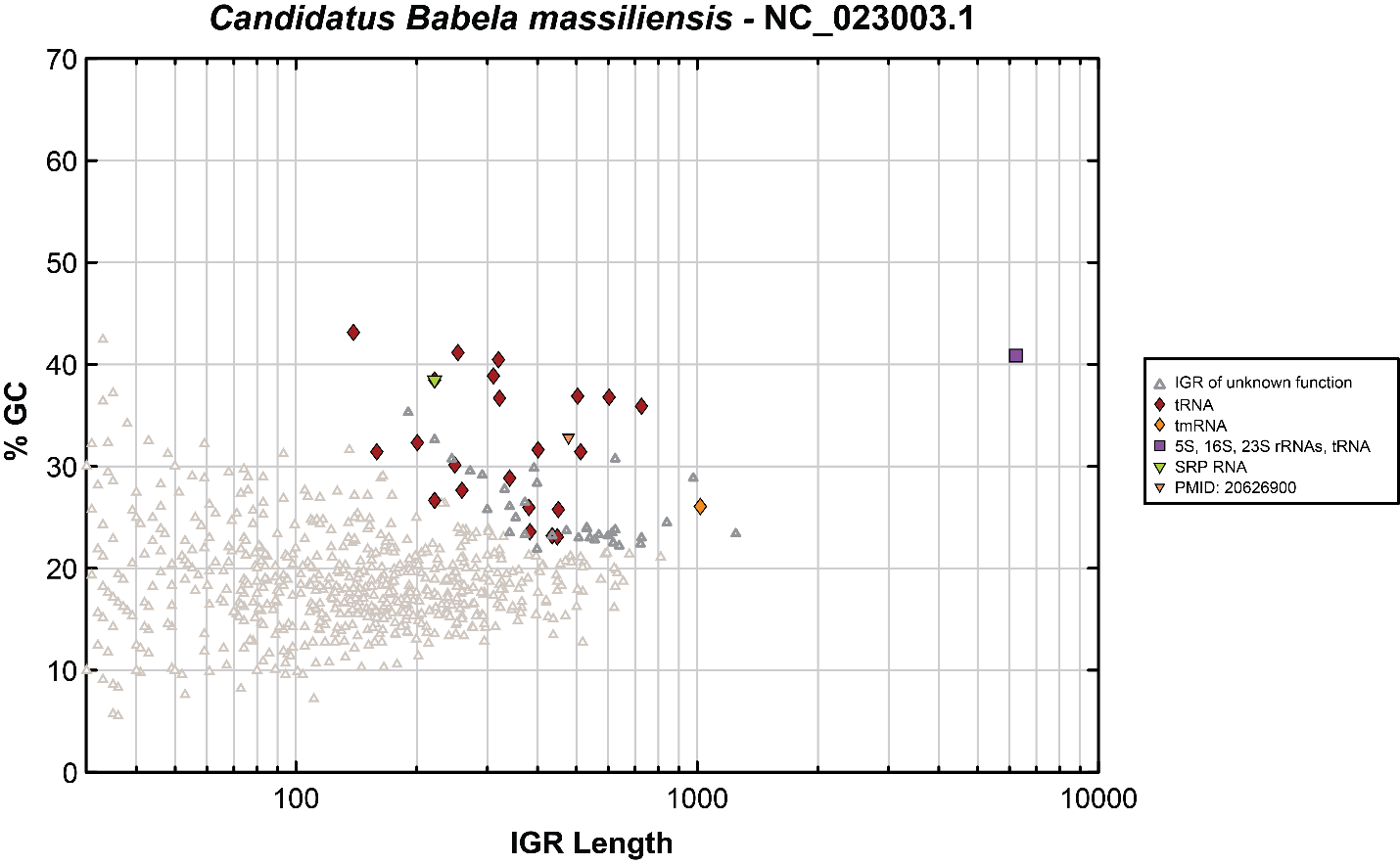


**Fig S26.** Plots of the IGRs from the *C. B. massiliensis* genome sorted by IGR length and GC content.

**
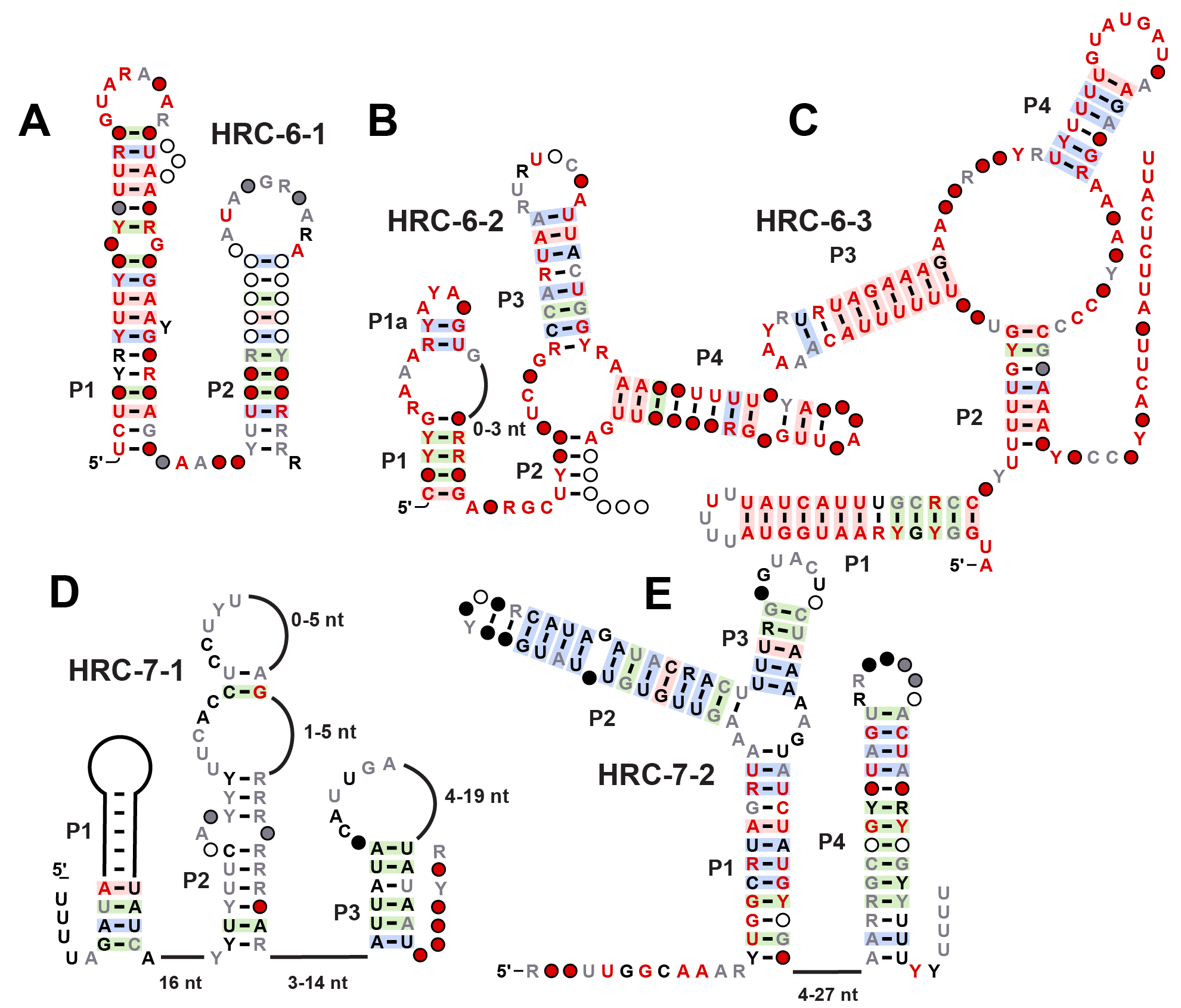
**

**Fig S27.** High-ranking candidates of unknown function from the genomes *L. monocytogenes* and *F. nucleatum*. The number of representatives and gene context are given in Supplementary File 1. (**A**) HRC-6-1 (**B**) HRC-6-2 (**C**) HRC-6-3 (**D**) HRC-7-1 (**E**) HRC-7-2

**
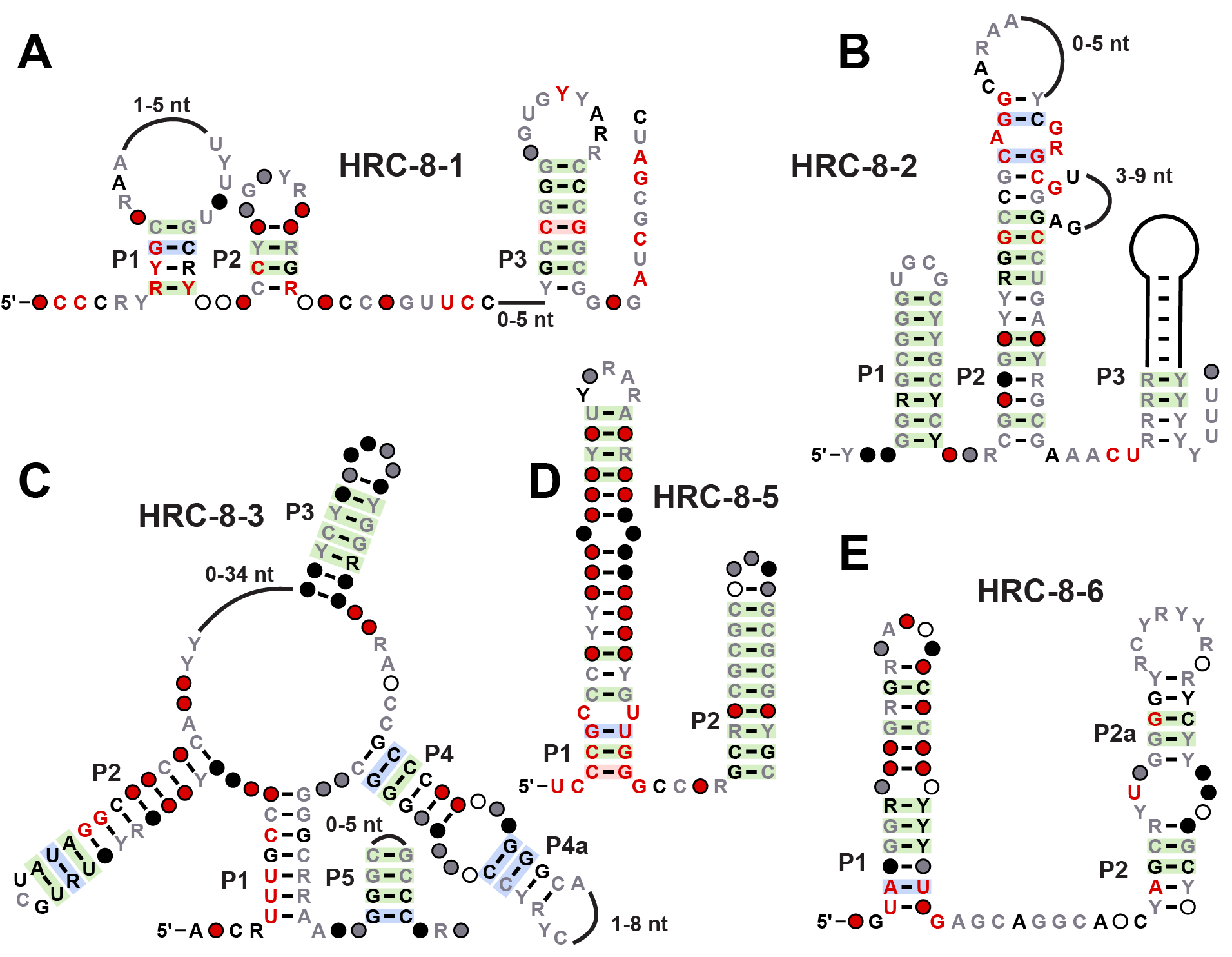
**

**Fig S28.** High-ranking candidates of unknown function from the genome *Ruegeria sp. TM1040*. The number of representatives and gene context are given in Supplementary File 1. (**A**) HRC-8-1 (**B**) HRC-8-2 (**C**) HRC-8-3 (**D**) HRC-8-4 (**E**) HRC-8-5 (**F**) HRC-8-6

**
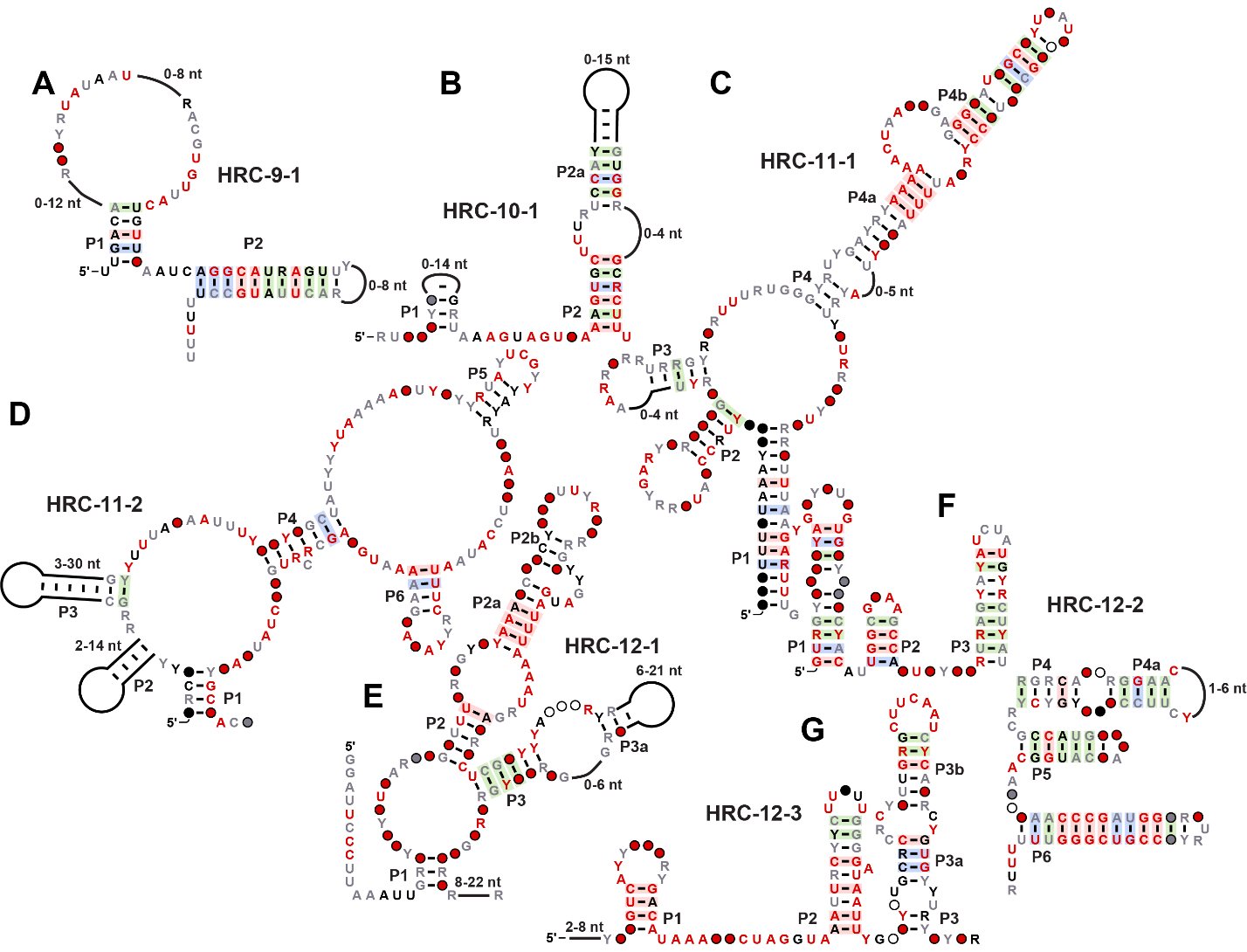
**

**Fig S29.** High-ranking candidates of unknown function from the genomes *C. perfringens* and *P. pentosaceus*, *C. fetus,* and *P necessarius*. The number of representatives and gene context are given in Supplementary File 1. (**A**) HRC-9-1 (**B**) HRC-10-1 (**C**) HRC-11-1 (**D**) HRC-11-2 (**E**) HRC-12-1 (**F)** HRC-12-2 (**G**) HRC-12-3


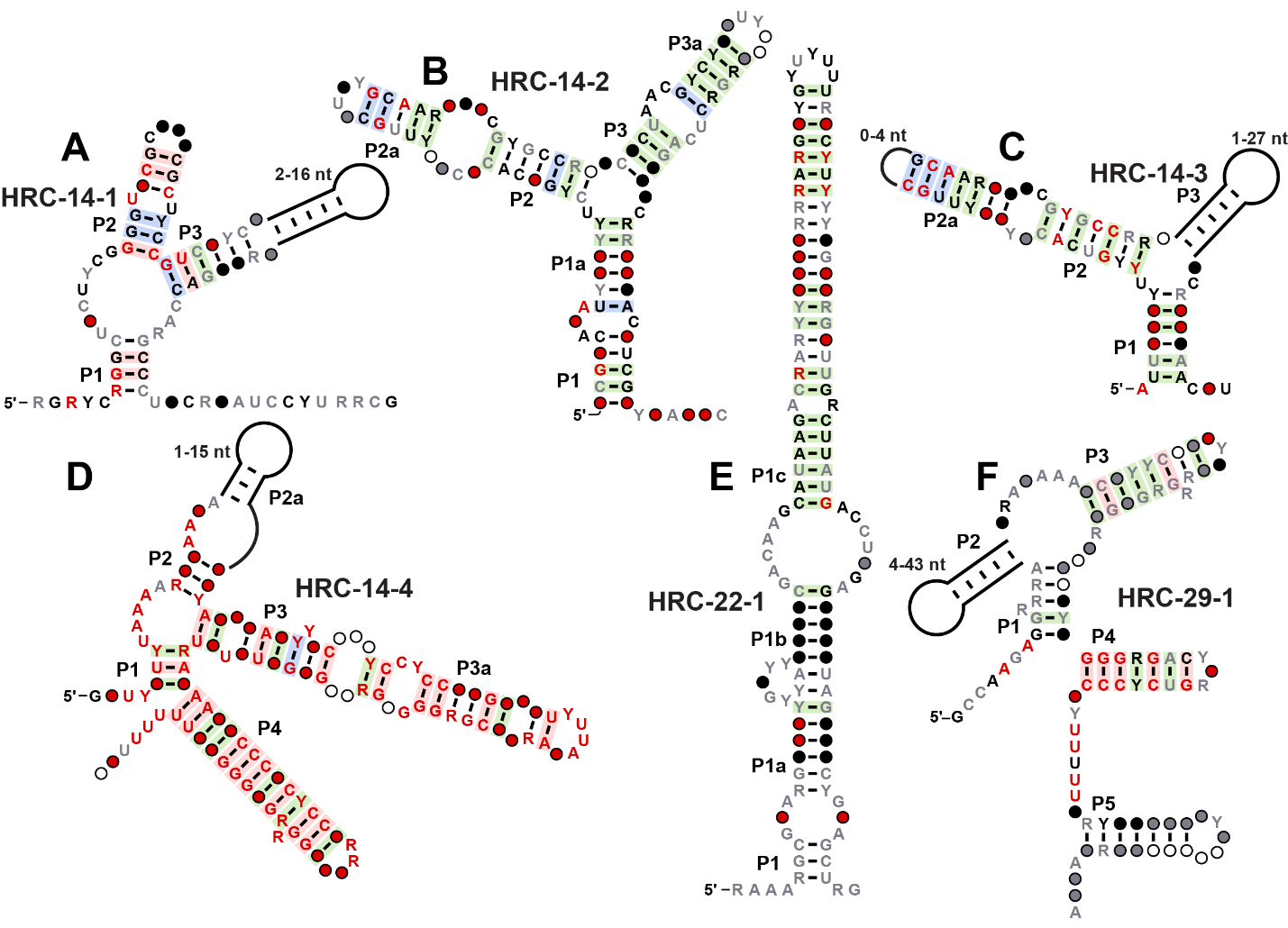


**Fig S30.** High-ranking candidates of unknown function from the genome *L. biflexa*, *H. maritima* and *S. salinus*. The number of representatives and gene context are given in Supplementary File 1. (**A**) HRC-14-1 (**B**) HRC-14-2 (**C**) HRC-14-3 (**D**) HRC-14-4 (**E)** HRC-22-1 (**F**) HRC-29-1

**
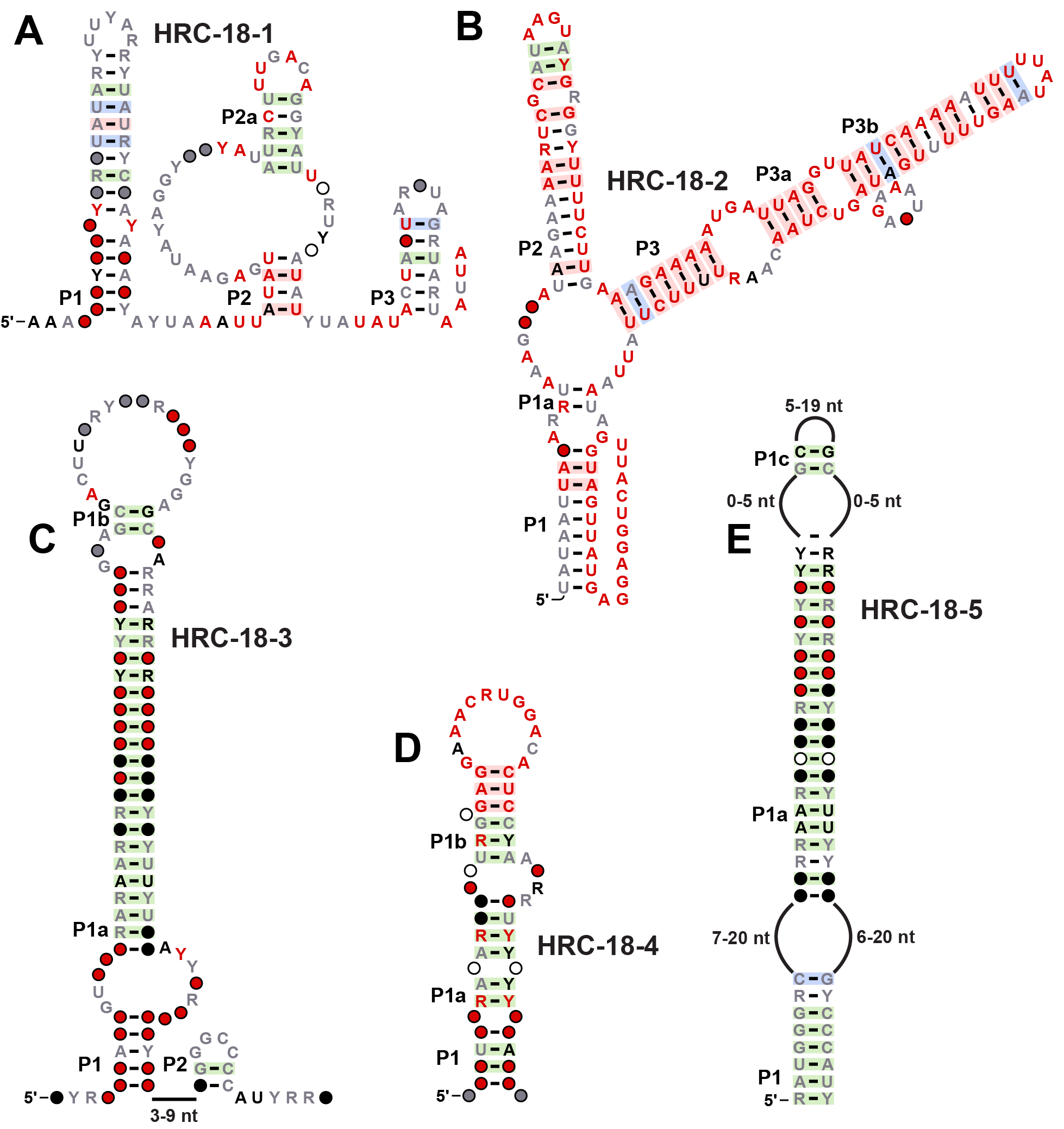
**

**Fig S31.** High-ranking candidates of unknown function from the genome *V. parvula*. The number of representatives and gene context are given in Supplementary File 1. (**A**) HRC-18-1 (**B**) HRC-18-2 (**C**) HRC-18-3 (**D**) HRC-18-4 (**E**) HRC-18-5

**
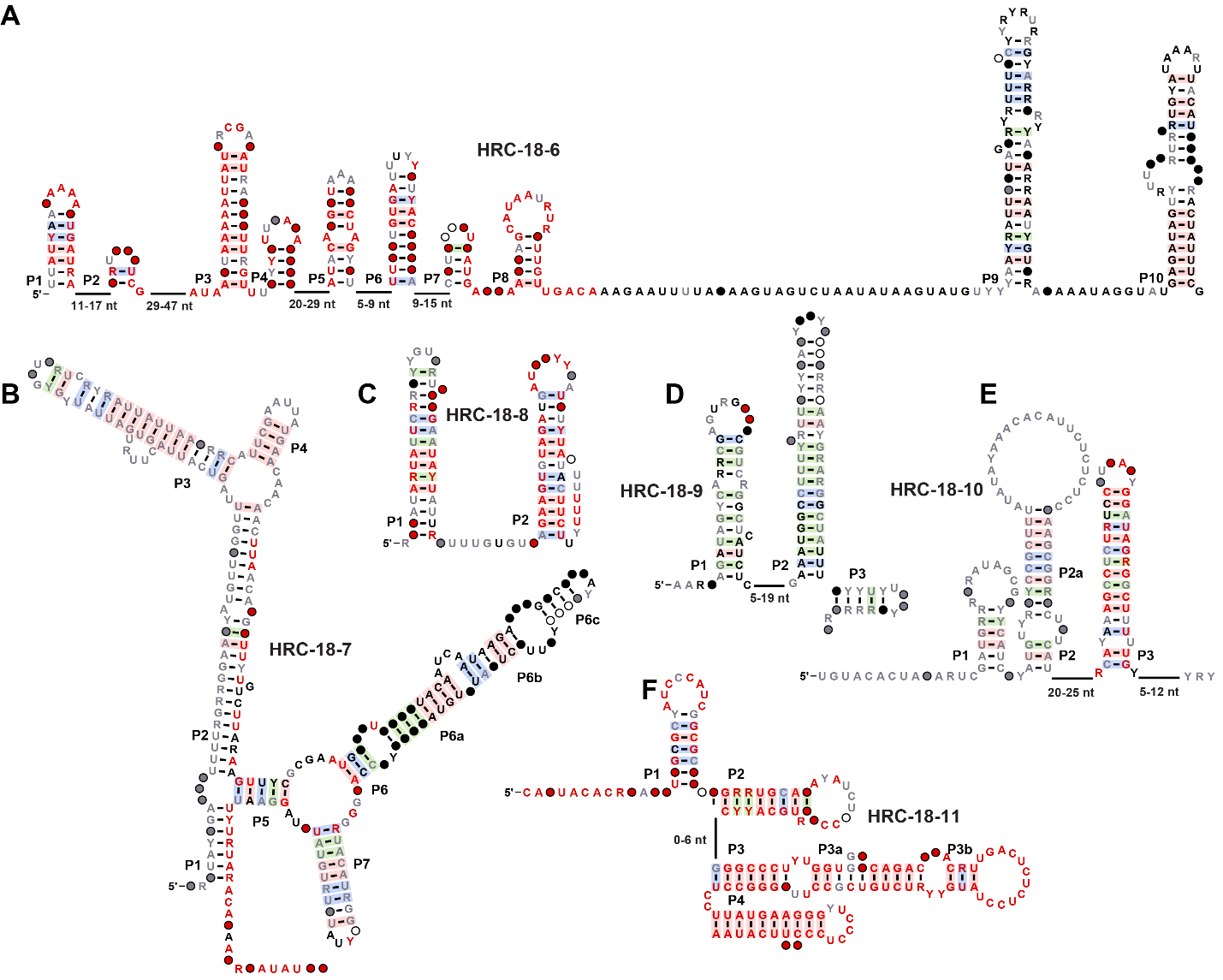
**

**Fig S32.** High-ranking candidates of unknown function from the genome *V. parvula* (cont.). The number of representatives and gene context are given in Supplementary File 1. (**A**) HRC-18-6 (**B**) HRC-18-7 (**C**) HRC-18-8 (**D**) HRC-18-9 (**E**) HRC-18-10 (**F**) HRC-18-11


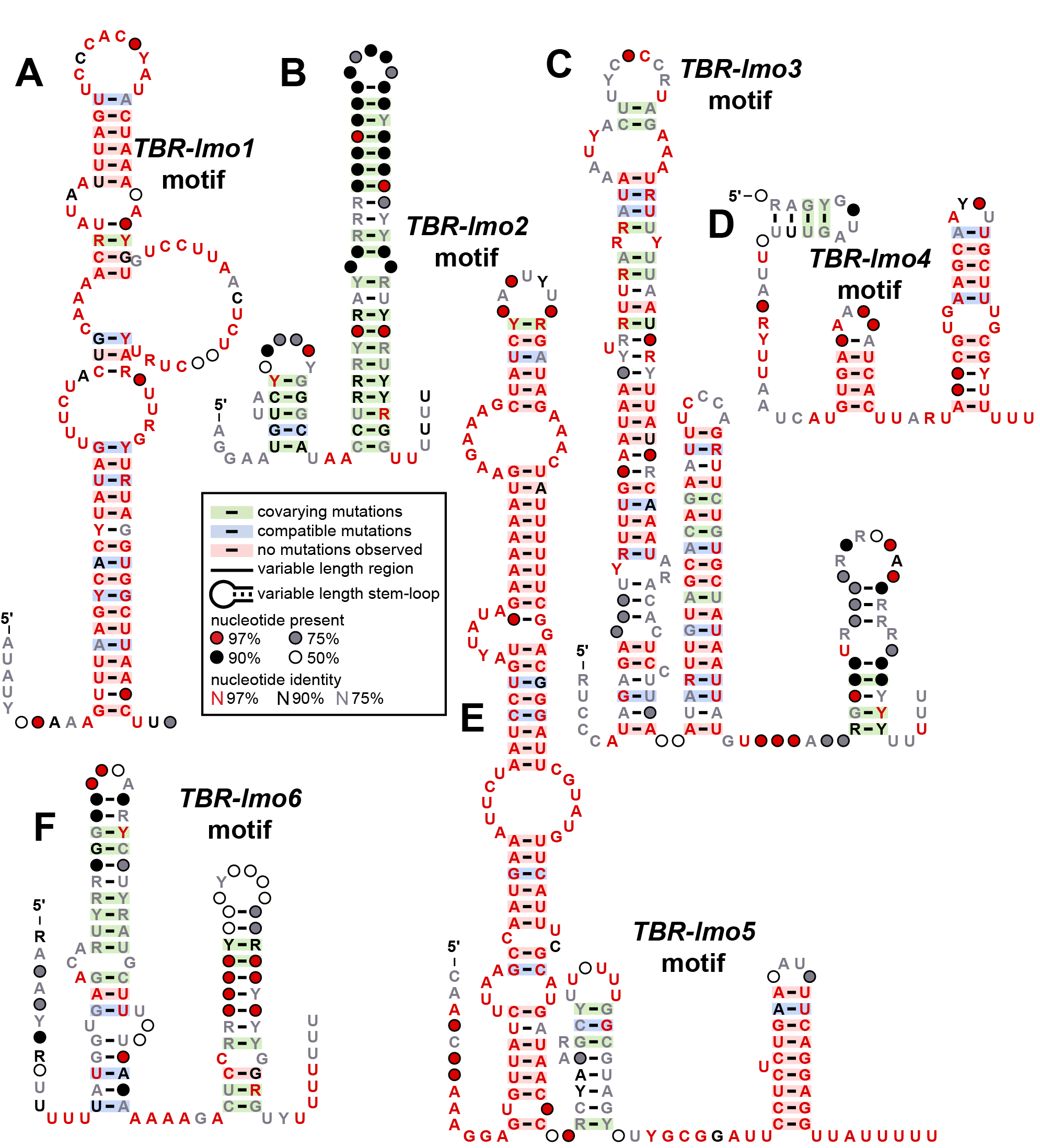


**Fig S33.** Motifs identified from the IGRs containing the “riboregulators” identified via term-seq [88]. The IGR for each riboregulator was added to the analysis pipeline despite having all, with one exception, been previously filtered out on the basis of IGR GC-content, length, or the presence of known structured RNAs. The most promising structured ncRNA motif resulting from the pipeline’s iterative cycle of Infernal search and CMFinder structure prediction is shown. (**A**) TBR-lmo1. This motif consists of 27 unique sequences found exclusively in various species of the *Listeria* genus. The structure appears to be an unremarkable terminator stem, although the predicted base-pairing is not supported by strong co-variation. The motif is found exclusively in front of genes coding for predicted Zn-dependent metalloproteases. (**B**) TBR-lmo2. This motif consists of 73 unique sequences found in several families of Bacillales. The structure is dominated by an intrinsic terminator stem that is strongly supported by co-variation. The motif can be found in front of collection of disaccharide-specific transporter and phosphatase encoding genes. (**C**) TBR-lmo3. This motif consists of 41 unique sequences found only in the *Listeria* genus. The structure consists of two adjacent stems moderately supported by covariation followed by a short terminator stem. The motif is with few exceptions found in front of genes encoding predicted divalent metal ion transporters. (**D**) TBR-lmo4. This motif consists of 31 unique sequences found only in the *Listeria* genus. The structure consists of three-particularly short stems poorly supported by covariation. Only the final stem appears to have length and stretch of U’s to indicate activity as a possible terminator. The motif is always found in front of predicted sulfate permease encoding genes. (**E**) TBR-lmo5. This motif consists of 25 unique sequences found only in the *Listeria* genus. The predicted structure consists of one long stem poorly supported by covariation, one short stem strongly supported by covariation and a short terminator stem. The motif is usually found near genes generically annotated as ABC transporter ATPase. (**F**) TBR-lmo6. This motif consists of 38 unique sequences found mostly in the *Listeria* genus. The predicted structure consists of two covariation supported stems, one of which is a clear terminator. The motif is always found upstream of genes encoding the uncharacterized membrane-anchored protein YitT.


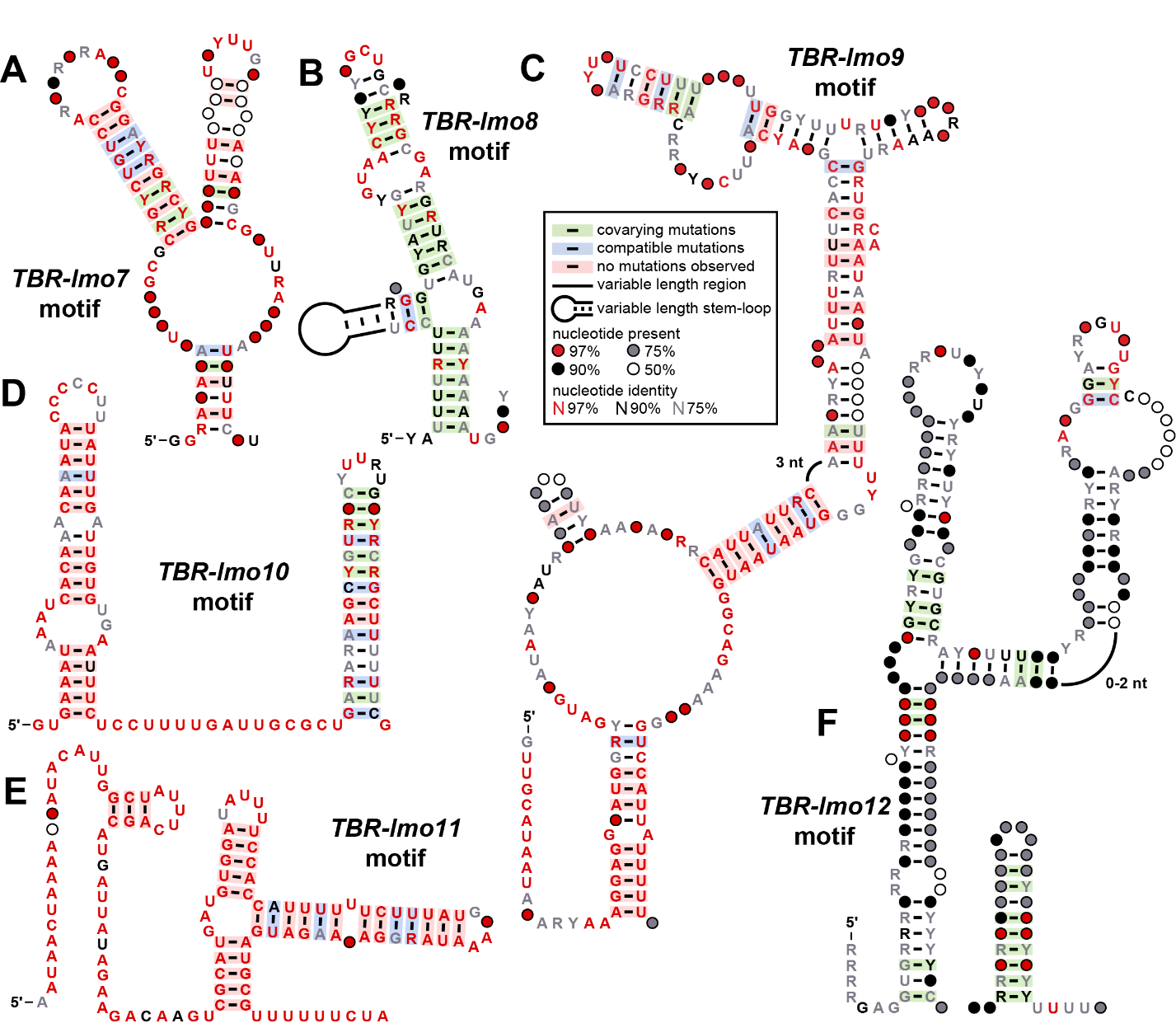


**Fig S34.** Additional motifs identified from the IGRs containing the “riboregulators” identified via term-seq [88]. Analysis performed as described in **Fig. S34**. (**A**) TBR-lmo7. This motif consists of 20 unique sequences found almost exclusively in the *Listeria* genus. The predicted structure for this motif is one of the few from this collection that contain a multi-stem junction. However, this structure has sonly some support from covariation. The motif is always found upstream of genes encoding acetyl-CoA carboxylase. (**B**) TBR-lmo8. This motif consists of 466 unique sequences found in a wide variety of Frimicutes. The genetic context upstream 30S of ribosomal protein S4 is indicative of a ribosomal leader candidate. (**C**) TBR-lmo9. This motif consists of 36 unique sequences found only in the *Listeria* genus. The structure of this motif is unusual with large loops in between co-variation supported stems and no clear terminator. This motif is typically found upstream of genes encoding putative membrane-bound multidrug transporters. (**D**) TBR-lmo10. This motif consists of 62 unique sequences found in multiple families of Bacillales. The structure of this motif consists of two simple stems one of which may be a terminator. This motif is nearly always found upstream of DUF3116, a domain of unknown function that appears to be restricted to Bacillales. (**E**) TBR-lmo11. This motif consists of 18 unique sequences found only in the genus *Listeria*. The narrow distribution of nearly identical sequences in this motif leave the predicted structure completely unsupported by covariation. There does, however, appear to be a plausible termination stem. . This motif is always associated with inosine 5'-monophosphate dehydrogenase. (**F**) TBR-lmo12. This motif consists of 359 unique sequences found in multiple families of Bacilli and Clostridia. Covariation strongly supports the presence of a terminator stem at the end of the motif, and there is some support from covariation for the three-stem multistem junction. This motif is always found upstream of genes encoding putative membrane-bound multidrug transporters.

**Supplementary File 1.** Supplementary File 1 contains an excel spreadsheet listing the coordinates for every IGR extracted and analyzed and the eventual categorization. Depending on the classification, additional data including number of representatives and genetic context is also reported.

**Supplementary File 2.** This file contains a zip archive with Stockholm alignments with all the novel motifs reported in this study.
